# Pre-COVID life expectancy, mortality, and burden of diseases for adults 70 years and older in Australia: a systematic analysis for the Global Burden of Disease 2019 Study

**DOI:** 10.1016/j.lanwpc.2024.101092

**Published:** 2024-06-05

**Authors:** GBD 2019 Australia Collaborators, Liliana G. Ciobanu, Nadezhda V. Baryshnikova, Magdalene Catharine Jawahar, Catherine G. Toben, Elysia Sokolenko, Victoria Kiriaki Arnet, Isaac Yeboah Addo, Oyelola A. Adegboye, Bright Opoku Ahinkorah, Khurshid Alam, Sheikh Mohammad Alif, Edward Kwabena Ameyaw, Deanna Anderlini, Blake Angell, Adnan Ansar, Anayochukwu Edward Anyasodor, Thomas Astell-Burt, Prince Atorkey, Beatriz Paulina Ayala Quintanilla, Getinet Ayano, Abraham Samuel Babu, Nasser Bagheri, Bernhard T. Baune, Dinesh Bhandari, Sonu Bhaskar, Soufiane Boufous, Andrew M. Briggs, Norma B. Bulamu, Richard A. Burns, Andre F. Carvalho, Ester Cerin, Nicolas Cherbuin, Enayet Karim Chowdhury, Marita Cross, Diego De Leo, Tim Robert Driscoll, Mi Du, David Edvardsson, Kristina Edvardsson, Ferry Efendi, Aklilu Endalamaw, Nelsensius Klau Fauk, Joanne Flavel, Richard Charles Franklin, Tiffany K. Gill, Bhawna Gupta, Vivek Kumar Gupta, Mohammad Hamiduzzaman, Graeme J. Hankey, Simon I. Hay, Jeffrey J. Hebert, Delia Hendrie, Catherine L. Hill, M. Mamun Huda, Sheikh Mohammed Shariful Islam, Billingsley Kaambwa, Himal Kandel, Gizat M. Kassie, Jessica A. Kerr, Asaduzzaman Khan, M. Nuruzzaman Khan, Vishnutheertha Kulkarni, Ratilal Lalloo, Long Khanh Dao Le, James Leigh, Gang Liu, Rashidul Alam Mahumud, Abdullah A. Mamun, John J. McGrath, Atte Meretoja, Ted R. Miller, Philip B. Mitchell, Ali H. Mokdad, Lidia Morawska, Kehinde O. Obamiro, Amy E. Peden, Konrad Pesudovs, Azizur Rahman, Md Mijanur Rahman, Muhammad Aziz Rahman, Zubair Ahmed Ratan, Lal Rawal, Susan Fred Rumisha, Perminder S. Sachdev, Abdul-Aziz Seidu, Saurab Sharma, Seyed Afshin Shorofi, Soraya Siabani, Ambrish Singh, Balbir Bagicha Singh, Helen Slater, Mark A. Stokes, Narayan Subedi, Santosh Kumar Tadakamadla, Amanda G. Thrift, Mai Thi Ngoc Tran, Corneel Vandelanotte, Ning Wang, Paul Ward, Mark Woodward, Xiaoyue Xu, Lalit Yadav, Sojib Bin Zaman, Jianrong Zhang, Scott Richard Clark

**Keywords:** GBD 2019, Global Burden of Disease Study, Australia, Aged 70 and above

## Abstract

**Background:**

The Australian population aged 70 and above is increasing and imposing new challenges for policy makers and providers to deliver accessible, appropriate and affordable health care. We examine pre-COVID patterns of health loss between 1990 and 2019 to inform policies and practices.

**Methods:**

Using the standardised methodology framework and analytical strategies from GBD 2019 methodologies, we estimated mortality, causes of death, years of life lost (YLLs), years lived with disability (YLDs), disability-adjusted life years (DALYs), life expectancy at age 70 and above (LE-70), and healthy life expectancy (HALE-70) in Australia comparing them globally and with high socio-demographic index (SDI) groups.

**Findings:**

DALY rates have been improving steadily over the past 30 years among Australians aged 70 and above. Decreases in DALY rates were primarily attributed to a fall in YLLs attributable to cardiovascular diseases (60%) and chronic respiratory disorders (30.2%) and transport injuries (56.9%), while the non-fatal burden remained stable from 1990 to 2019. According to the DALY rates, the top five leading causes are ischemic heart disease, Alzheimer's disease, COPD, stroke, and falls, where falls exhibited the largest increase since 1990.

**Interpretation:**

This study provides an in-depth report on the main causes of mortality and disability in Australia's population aged 70 and above. It sheds light on the shifts in burden over three decades, emphasising the need for the Australian health system to enhance its readiness in addressing the escalating demands of an ageing population. These findings establish pre-COVID baseline estimates for Australia's population aged 70 and above, informing healthcare preparedness.

**Funding:**

Bill & Melinda Gates Foundation.

**Research in context**

**Evidence before this study**

Australia's healthcare system is globally renowned for providing high-quality care to individuals aged 70 and over. With some of the highest life expectancies and a high quality of life on a global scale, Australia's commitment to the well-being of its senior citizens is evident. However, it is essential to acknowledge the substantial increase in per capita health expenditure over recent decades, which currently ranks among the highest worldwide. The advent of COVID-19 has added new challenges to the Australian healthcare system, particularly affecting one of the most vulnerable populations: those aged 70 and over.

To maintain the delivery of effective and high-quality care and to guide preventative measures for the well-being of the 70 and above population, it is crucial to understand the burden of disease for this demographic and how these trends have evolved over time. The Australian Institute of Health and Welfare introduced the Australian Burden of Disease Study (ABDS) through a series of reports (1996, 2003, 2011, 2015, 2018), with the most recent one published in 2022. These reports aimed to assess the burden of disease in Australia and compare it with previous findings. However, it is important to note that the ABDS 2022 study solely provided point-in-time estimates for 220 conditions, offering an incomplete perspective on temporal trends. Furthermore, this study did not specifically analyse the population aged 70 and over, overlooking a critical demographic. Additionally, these individual studies did not account for potential confounding factors or differences across different study periods. Lastly, these estimates are not directly comparable with international studies due to variations in methodology. To address these limitations and enhance the depth of our understanding, our study relies on standardised, globally recognised estimates sourced from the Global Burden of Disease 2019 Study.

**Added value of this study**

Our study represents a comprehensive analysis of the disease burden in the Australian population aged 70 and over, employing standardised and globally comparable metrics. We provide a thorough assessment of life expectancy, mortality, and the burden of diseases for adults aged 70 and older in Australia from 1990 to 2019, offering a nuanced and internationally comparable perspective on the health of Australians aged 70 and over. This study establishes crucial pre-COVID baseline estimates for Australia's population aged 70 and over, thereby informing healthcare preparedness.

**Implications of all the available evidence**

Estimates of disease burden in Australia serve as invaluable evidence to guide population health policies and prioritise healthcare initiatives. As life expectancy at birth steadily rises and mortality rates decline, the burden of disease in Australia increasingly encompasses functional health challenges associated with aging. This includes the top five leading causes of DALYs: ischemic heart disease, Alzheimer's disease, COPD, stroke, and falls, with falls exhibiting the most significant increase since 1990. To ensure the long-term sustainability of the Australian healthcare system, it is imperative to implement preventive and remedial healthcare policies and practices that specifically target these pressing issues.

## Introduction

Older people account for an increasing share of the total Australian population.^1^ Over the 20 years between 1999 and 2019, the proportion of the Australian population 65 years and over increased from 12.3% to 15.9%, which is higher than the global estimate of 9%.^2^ Ageing of the population puts unsustainable pressure on public spending and subsidised health care,^3^, ^4^, ^5^ which was exacerbated significantly by the COVID-19 pandemic.^6^

Several studies reporting that Australian health systems falls short when it comes to providing adequate care for older Australians^7^ especially for culturally and linguistically diverse populations^8^ as well as in rural areas, where waiting times for medical care are long.^9^ The common occurrence of multimorbidity in older individuals^8^^,^^10^, ^11^, ^12^ and the challenge of aligning financial incentives in a mixed public-private health care and financing system,^9^ further amplifies the difficulty in accessing treatment. In acknowledgement of this need the Australian government has recently announced new policy commitments to strengthen primary health care for the elderly. This includes significant local investment in a new chronic disease care funding model for those aged 70 years and above ($448.5 million over two years from 2020 to 21) and the Practice Incentives Program Quality Improvement Initiative, including retention of the Aged Care Access Incentive ($201.5 million over five years) and the Dementia, Aging and Aged Care Mission ($167.5 million over 10 years starting in 2019–2021). The research spending on aged health has been allocated $45.9 million dollars in 2019 up from $32.1 million in 2013 (NHMRC).

For appropriate policy planning and targets for research, detailed timely updates on disease burden and expenditure by age and by disease, are critical. Such data is particularly important as a baseline pre the impact of the coronavirus 2019 (COVID-19) pandemic, where older adults had the highest risk of severe disease, hospitalisation, and death.

Currently, the main source for Australian Government health-related budget allocations and decision making are the estimates produced by The Australian Institute of Health and Welfare (AIHW), which introduced the Australian Burden of Disease Study (ABDS) through a series of reports in 1996, 2003, 2011, 2015, and 2018. While the ABDS provides a coverage of burden estimates at sub-national level, the most recent 2022 estimates for 220 conditions^13^ providing incomplete information about trends over time, which are also not directly comparable with those produced earlier or by other institutions (mainly, due to differences in methodology and applied data resources). These limitations affect timely decision making.

The Global Burden of Disease Study 2019 offers consistent country-specific disease burden data, enabling cross-location and time point comparisons.^14^ With yearly data since 1990 and international collaboration, it aids between-country assessments and modelling for local insights. In this study, our primary objectives are to analyse Australia's GBD 2019 estimates, focusing on the 70 years and above population. We aim to track trends and make comparisons with the estimates for high Socio-Demographic Index (SDI) countries group, across key GBD metrics.^14^ These findings are crucial for timely, evidence-based insights into health loss among the 70 and above population, facilitating policy impact assessment, effective prevention and treatment strategies, and healthcare resource planning.

## Methods

### Overview

We analysed data of the GBD 2019 study to evaluate Australian trends in epidemiological patterns and disease burden for people aged 70 years and older for 1990–2019. The analyses employed established GBD 2019 summary measures of morbidity and mortality, including death rates, years of life lost (YLLs), years lived with disability (YLDs), disability-adjusted life years (DALYs), life expectancy at 70 (LE-70), and health adjusted life-expectancy aged 70 (HALE-70). Seventy years and older is the GBD cut-off used to define old age. This threshold was selected based on literature (a historical threshold reflecting the Social Security Act, 1991),^15^ the availability of pre-determined age groups in the GBD study, and to provide comparable findings to the majority of studies on older people. Current GBD estimates are available in public domain and can be accessed: https://vizhub.healthdata.org/gbd-compare/. This manuscript adheres to the Guidelines on Accurate and Transparent Health Estimate Reporting.^16^

### Input data sources

Input data were extracted from censuses, household surveys, civil registration and vital statistics, disease registries, health service use, disease notifications, health surveys, administrative databases, and other sources. The Global Health Data Exchange, GHDx, source tool is publicly available to identify a complete list of 1020 data sources used for estimating any disease or injury outcome in Australia (https://ghdx.healthdata.org/gbd-2019/data-input-sources). (Full list in Supplementary Table S1).

### GBD measures

Below, we provide a concise overview of each measure of the GBD 2019. For a comprehensive understanding of the methods employed in the GBD demographic estimation process, detailed descriptions can be found in previous publications.^14^^,^^17^^,^^18^ In the GBD 2019 study, causes of mortality and morbidity were organised into a levelled cause hierarchy from the broadest causes of death and disability at Level 1 to the most specific causes at Level 4 defining 369 diseases and injuries, which were aggregated in three Level 1 causes, 22 Level 2 causes, 174 Level 3 causes, and 301 Level 4 causes. In total, 364 total causes are non-fatal and 286 are fatal. The full GBD cause hierarchy, including corresponding International Classification of Diseases (ICD)-9 and ICD-10 codes, is published in detail previously.^14^

Only the top 20 causes of deaths, YLLs, YLDs, and DALYs, selected based on the GBD ranking estimates, are presented in our analysis. In the Results section, we used the Level 3 cause categorisation, providing a balance between specificity and comprehensiveness, unless otherwise specified.

Cause-specific death rates and cause fractions were derived using advanced statistical techniques, namely the Cause of Death Ensemble model and spatiotemporal Gaussian process regression. To ensure accuracy, cause-specific deaths were adjusted to align with the total all-cause deaths calculated in the broader GBD framework, which includes population, fertility, and mortality estimates. Years of life lost (YLLs), defined as the number of years of life lost due to premature death, defined as dying before reaching the estimated life expectancy for a specific population, were computed by multiplying the adjusted cause-specific deaths by the standard life expectancy at each age.

To maintain consistency across various measures, a Bayesian meta-regression modelling tool DisMod-MR 2.1 was employed. This tool facilitated the harmonisation of incidence, prevalence, remission, excess mortality, and cause-specific mortality for most causes. By multiplying prevalence estimates with disability weights^19^ assigned to distinct disease sequelae and injuries, years lived with disability (YLDs), defined as one YLD is one year of health life lost, were estimated. This approach ensured the inclusion of the impact of different health conditions on overall disability burden.

Disability-adjusted life years (DALYs), a measure of healthy life lost due to premature death or living with disability, were calculated by summing YLDs and YLLs for each disorder. One DALY equates to one lost year of healthy life.

Life expectancy at age 70 (LE-70), is defined as the number of years a person can expect to live once he/she reaches a 70-year-old mark, and was estimated based on expected mortality rates using the meta-97 regression tool MR-BRT (meta-regression-Bayesian regularised trimmed) to analyse the relationship between log mortality rates and SDI.^14^^,^^20^ Healthy adjusted life expectancy (HALE-70) is an estimation of the number of remaining healthy years a 70-year-old can expect to live. It serves as a summary metric for both the age-specific mortality and morbidity for a given population in a calendar year. We followed the analytical methods used to generate HALE in the GBD 2017 cycle.^21^

### Uncertainty intervals (UI)

For all results, we report 95% uncertainty intervals (UIs) derived from 1000 draws from the posterior distribution of each step in the estimation process according to the established GBD methodology. We present sex-specific rates per 100,000 population aged 70 and above, and percentage changes from 1990 to 2019 for deaths, YLLs, YLDs, DALYs, and deaths for 20 top causes.

### Comparison with high socio-demographic index GBD group

Socio-demographic Index (SDI) is a composite indicator of development status strongly correlated with health outcomes. It is the geometric mean of 0–1 indices of total fertility rate under the age of 25 (TFU25), mean education for those ages 15 and older (EDU15+) and lag distributed income (LDI) per capita. It is expressed on a scale of 0–1, with 0 being the lowest SDI value and 1 being the highest. The high SDI group in 2019 consists of 38 countries (including Australia) with the SDI index values above 0.805. The list of high SDI countries and their yearly SDI values is provided in the Supplementary Table S2. We compared the burden of disease in Australia with the average of high SDI group and global estimates.^22^

### Role of the funding source

The GBD is funded by the Bill & Melinda Gates Foundation and the National Institute on Ageing of the National Institutes of Health. SC was supported by Janssen-Cilag Australia and Lundbeck Otsuka grants paid to institution (The University of Adelaide). These funders had no role in study design; collection, analysis, and interpretation of data; or writing of the manuscript. All authors had full access to the data in the study and had final responsibility for the decision to submit for publication.

## Results

### Life expectancy (LE-70) and healthy adjusted life expectancy at age 70 and above (HALE-70), mortality at ages 70 and above, and decomposition of causes of death

Over the past thirty years, life expectancy at age 70 (LE-70) in Australia has risen for both males and females. In 1990, males had a LE-70 of 12.1 years (95% UI: 12.0–12.1), which increased to 16 years (95% UI: 15.8–16.1) in 2019. For females, LE-70 went from 15.3 years (95% UI: 15.3–15.4) in 1990 to 18.3 years (95% UI: 18.2–18.4) in 2019. This narrowing gap between males and females decreased from 3.2 years in 1990 to 2.3 years in 2019. When considering years lived in poor health, HALE-70 in 2019 showed little difference between males (11.1 years (95% UI: 9.9–12.1)) and females (12.4 years (95% UI: 11–13.6)). However, the annual percent change from 1990 to 2019 was higher for males (0.88%) than females (0.51%), suggesting a narrowing gender gap in LE-70 and HALE-70 in Australia, consistent with global patterns. Decomposition analysis of the increase in Life Expectancy (LE) from 1990 to 2019 indicates that the top three causes contributing to the LE increase are cardiovascular diseases (+3.5 years), neoplasms (+0.9 years), and transport injuries (+0.4 years). Substance use, on the other hand, contributes to a decrease in LE (−0.1 years). For more detailed information, refer to Supplementary Table S3.

Despite a 75.9% increase in the absolute number of deaths among those aged 70 and above in Australia from 1990 to 2019, reflecting population growth and aging, the all-cause mortality rate decreased by 24% from 6097 deaths per 100,000 (95% UI: 6065–6129) in 1990 to 4636 deaths per 100,000 (95% UI: 4563–4713) in 2019. This reduction was more pronounced in males (30.9%) compared to females (18.8%), consistent with a global decrease of 20.5% (22.5% in males and 19.7% in females) for the same period. Simultaneously, there is an overall LE-70 increase of 0.74% in this pattern from 1990 to 2019. However, it's noteworthy that the percentage change has decreased over the past decade, with a 0.97% increase in 1990–2000, a 0.92% increase in 2000–2010, and only a 0.29% increase in 2010–2019.

Cardiovascular diseases remained the leading cause of death in 2019, with 1594 deaths per 100,000 (95% UI: 1336–1737) for both sexes, despite a 49.7% decrease from 1990 when it was 3172 deaths per 100,000 (95% UI: 2891–3313). However, certain causes saw a steady increase over time. Deaths due to falls and chronic kidney disease (CKD) increased by 105.1% and 44.9%, respectively, from 1990 to 2019. Falls accounted for 57.3 deaths per 100,000 (95% UI: 49.9–63.2) in 1990 and 117.6 deaths per 100,000 (95% UI: 95.7–133) in 2019, while CKD was responsible for 115.4 deaths per 100,000 (95% UI: 103.3–124.3) in 1990 and 167.2 deaths per 100,000 (95% UI: 135.1–191) in 2019.

Sex-specific trends in causes of death reveal consistent, significant increases in deaths attributed to CKD (F: 46.2%, M: 41.6%) and Falls (F: 101.2%, M: 113.2%). Conversely, ischemic heart disease (F: −58.1%, M: −62%) and stroke (F: −42.8%, M: −43.3%) witnessed substantial declines from 1990 to 2019. Lung cancer exhibited a noteworthy sex-specific divergence in trends, with a surge in females (36.9%) and a decline in males (−33.8%) (Table 1).

**Table 1:** The 20 top sex-specific leading causes of deaths (Level 3) in Australia from 1990 to 2019.

Deaths per 100,000											
Females						Males					
Cause of death (rank 2019)	1990	2000	2010	2019	% change (1990–2019)	Cause of death (rank 2019)	1990	2000	2010	2019	% change (1990–2019)
Ischemic heart disease	1767.20 (1556.5–1877.2)	1299.60 (1100.3–1406.1)	904.05 (723.9–1004.5)	741.15 (579.5–836.2)	−58.1∗	Ischemic heart disease	2318.04 (2190.2–2403.4)	1590.66 (1471.5–1664.2)	1051.31 (938.9–1116.6)	881.40 (772.8–952.3)	−62∗
Alzheimer's disease	352.09 (88.2–930.2)	409.03 (105.2–1058.1)	489.69 (128.6–1204.2)	476.11 (126–1175.4)	35.2	Stroke	737.34 (684.2–780.2)	584.68 (531.3–620.9)	422.93 (374–455.9)	364.64 (309.6–309.3)	−50.5∗
Stroke	793.03 (683.4–849.9)	652.23 (546.4–712.8)	532.66 (427.8–592.7)	453.28 (358.1–515.4)	−42.8∗	COPD	600.88 (552–641.9)	442.59 (399.3–483)	354.37 (307.6–403.4)	340.83 (279.6–409.9)	−43.3∗
COPD	193.28 (165.3–230.1)	223.58 (178.1–253.6)	233.93 (169.8–275.1)	232.93 (161.2–289.4)	20.5	Lung cancer	463.08 (438.3–484)	401.70 (377.6–419.5)	357.25 (327.1–377)	306.55 (275.1–335.6)	−33.8∗
Lung cancer	121.31 (110.7–128.9)	150.71 (134.4–161.4)	174.06 (147.8–189.4)	166.12 (139.8–187.1)	36.9∗	Prostate cancer	355.17 (269.5–427.5)	346.65 (273.3–434.04)	330.52 (269.1–448.7)	302.64 (245.4–427.6)	−14.8
Chronic kidney disease	109.85 (95.6–119.9)	119.02 (99.5–131.5)	157.18 (125.4–176.6)	160.58 (124.1–188.6)	46.2∗	Alzheimer's disease	193.39 (44.6–548.2)	214.51 (51.2–591.7)	265.44 (64.5–725.1)	287.53 (70.6–769.4)	48.7
Colon and rectum cancer	181.80 (162.7–193.9)	170.78 (149.2–184.4)	154.08 (129.2–170)	151.08 (124.8–172)	−16.9	Colon and rectum cancer	243.52 (229.1–256.2)	222.02 (207.4–234.4)	195.64 (178.7–207.8)	185.31 (161.9–208.3)	−23.9∗
Lower respiratory infections	109.64 (92.2–120.5)	145.98 (121.9–161.5)	127.27 (100.1–142.9)	145.60 (111.4–168.8)	32.8	Chronic kidney disease	123.72 (112.6–132.8)	138.84 (125.9–149.4)	165.68 (147–180)	175.16 (175.2–150.7)	41.6∗
Atrial fibrillation and flutter	116.74 (89–129.8)	133.59 (102.1–151.9)	144.83 (108.4–179.4)	139.75 (105.8–179.4)	19.7	Diabetes mellitus	136.45 (125.6–146.4)	158.84 (147.4–169.6)	165.62 (149–177.9)	136.65 (118.7–153.1)	0.1
Breast cancer	146.18 (131.5–155.8)	134.89 (117.9–144.4)	134.92 (113–147.8)	130.72 (106–148.3)	−10.6	Lower respiratory infections	128.58 (116.5–138.7)	153.38 (137.2–165)	119.64 (105.6–130.4)	129.82 (109.8–145.9)	1.0
Falls	59.87 (50.6–67.6)	74.02 (60.9–84.2)	115.29 (91.6–132.4)	120.46 (93.4–141.4)	101.2∗	Parkinson's disease	95.28 (88.6–100.5)	108.43 (99.1–114.4)	121.55 (108.7–129.2)	114.54 (100–124.9)	20.2
Diabetes mellitus	116.79 (103.1–126.8)	118.97 (102.1–129.9)	141.33 (114.4–157.8)	110.67 (88.9–127.1)	−5.2	Falls	53.54 (47.9–59.2)	66.11 (59–72.6)	108.36 (94–120.3)	114.16 (95.8–129.5)	113.2∗
Pancreatic cancer	63.72 (57.5–68.1)	71.09 (62.8–76.4)	78.32 (65.9–86.3)	80.47 (66.8–92.5)	26.3	Atrial fibrillation and flutter	68.78 (44–89)	77.76 (52.8–103.2)	88.17 (59.6–116.9)	90.05 (59.2–118.2)	30.9
Non-rheumatic valvular heart disease	51.81 (44.6–56.8)	48.38 (40–53.9)	58.63 (45.9–66.5)	55.83 (43–66.5)	7.7	Pancreatic cancer	76.85 (71.6–81.4)	75.95 (70.7–80.4)	87.12 (79.5–93)	88.07 (77.5–99.6)	14.6
Parkinson's disease	45.58 (40.2–49.1)	51.63 (44.4–55.8)	56.65 (46.9–62.4)	51.85 (41.2–57.9)	13.8	Leukemia	64.85 (60.2–69.4)	60.61 (55.4–64.9)	60.78 (54.8–65.6)	63.92 (53.7–74)	−1.4
Hypertensive heart disease	48.77 (31.3–55.1)	42.49 (31.5–52.5)	49.17 (33.1–58.3)	50.12 (33–61.2)	2.8	Bladder cancer	74.12 (69.6–78.7)	67.74 (62.6–71.6)	66.03 (59.5–70.6)	62.98 (54.4–71.4)	−15.0
Endo/metab/blood/immune disorders	15.55 (12.8–19.8)	28.39 (19.5–32.4)	48.88 (26.9–56.4)	48.09 (27.4–58.8)	209.3∗	Non-Hodgkin lymphoma	64.75 (58.6–71)	70.46 (64.4–76.8)	63.82 (57.3–69.8)	58.86 (48.7–71.3)	−9.1
Urinary diseases	25.02 (21.5–27.9)	39.71 (31–44.6)	40.33 (31.1–46.4)	47.51 (35.5–56.7)	89.9∗	Non-rheumatic valvular heart disease	52.35 (47.3–56.6)	44.94 (40–48.9)	55.57 (45–61.1)	57.14 (47.9–65.4)	9.2
Ovarian cancer	45.47 (39.7–49.2)	48.80 (42.8–53)	46.64 (39.3–52.9)	44.75 (36.5–54.1)	−1.6	Esophageal cancer	50.14 (46.7–53.3)	58.40 (52.2–62)	57.25 (52.5–61.2)	56.48 (48.7–64.5)	12.6
Peripheral artery disease	28.76 (9.3–57.6)	35.92 (12.7–76.1)	42.37 (15.6–95.6)	42.72 (16–96.8)	48.6	Stomach cancer	98.14 (92.8–103.4)	74.42 (69.1–78.5)	59.71 (54.6–63.6)	52.61 (46.5–58.6)	−46.4∗

Percent change (% change) of statistical significance is marked by an asterisk (∗).

For a comparison of the top 20 sex-specific causes of death between global estimates, Australia, composite scores of the ‘High SDI' group of 38 countries, and selected 14 individual countries, please refer to Supplementary Figures S1 and S2.

### Leading causes of YLLs among those aged 70 and older

In the last three decades, Australia witnessed a substantial 35.6% decrease in total fatal burden among adults aged 70 and above, declining from 78,550 (95% UI: 78,131–78,984) YLLs per 100,000 in 1990 to 50,585 (95% UI: 49,729–51,502) YLLs per 100,000 in 2019. This remarkable decline was primarily driven by reductions in YLLs attributed to cardiovascular diseases (−60%), chronic respiratory diseases (−30.2%), and transport injuries (−56.9%) (Level 2), collectively accounting for 39.5% of the total fatal burden in 2019. The leading cause of Level 2 fatal burden in Australia in 2019 was neoplasms, followed by cardiovascular and neurological diseases (particularly Alzheimer's, a Level 3 cause), constituting 73.3% of the total fatal burden.

Ischemic heart disease remained the primary cause of Level 3 fatal burden among older adults in Australia for nearly three decades, despite a decrease in YLLs from 25,435 per 100,000 (95% UI: 23,61–26,412) in 1990 to 8063 YLLs per 100,000 (95% UI: 6908–8764) in 2019. These estimates aligned with global trends, with 2019 YLLs lower than those for high SDI countries (9423 per 100,000; 95% UI: 8268–10,085), although significantly lower than global estimates (15,459 per 100,000; 95% UI: 14,061–16,465).

**Fig. 1:**
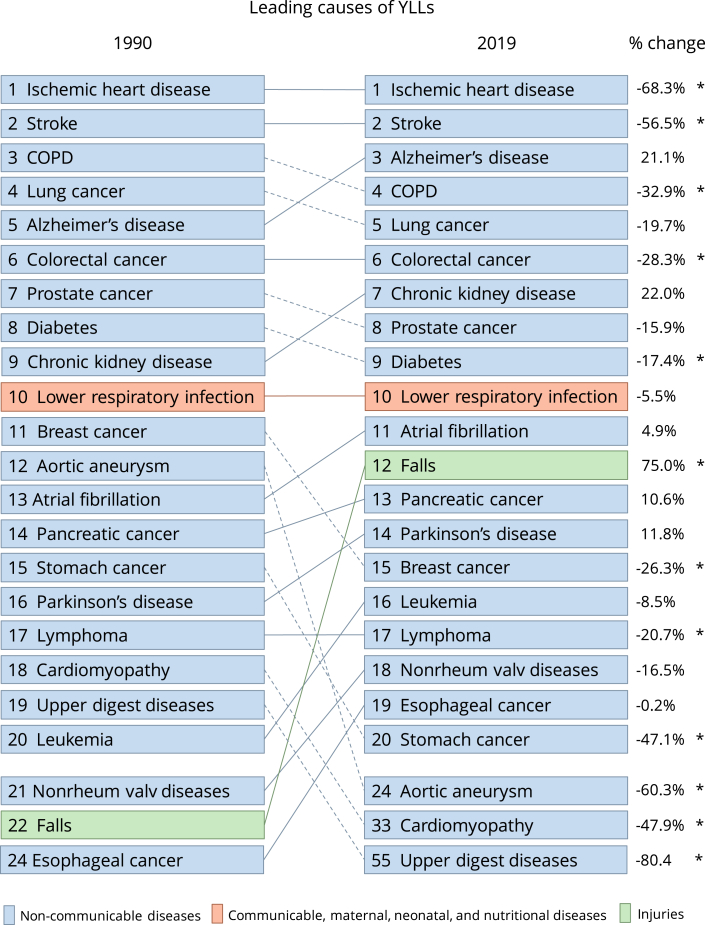
The top causes (Level 3) and % change of YLLs for both sexes combined in Australia 1990–2019. Dotted lines show a decrease in rank and solid lines show an increase in rank. Percent change (%change) of statistical significance is marked by an asterisk (∗). Abbreviations: Nonrheum valv diseases, Non-rheumatic valvular health diseases; Upper digest diseases, Upper digestive system diseases.

Consistent with trends for ischemic heart disease, the YLLs rate significantly decreased for stroke, COPD, colorectal cancer, diabetes, breast cancer, lymphoma, stomach cancer, aortic aneurysm, cardiomyopathy, and upper digestive diseases from 1990 to 2019. In 2019, several sex-specific differences emerged: fatal burden attributable to COPD, lung cancer, colorectal cancer, diabetes, lymphoma, and stomach cancer was notably higher in males than females. However, no sex-based difference was observed in YLLs attributable to stroke. The decline in YLLs rate for stroke, COPD, breast cancer, and stomach cancer in Australia from 1990 to 2019 mirrored global estimates (Fig. 1).

The YLLs rate attributed to falls in Australia surged by 75% from 1990 (630.4 YLLs per 100,000; 95% UI: 562.1–689.7) to 2019 (1103 YLLs per 100,000; 95% UI: 916.1–1236.4). This increase exhibited similar patterns for both males and females. Notably, these Australian estimates for 2019 were not significantly different from the global (1145.8 YLLs per 100,000; 95% UI: 935.7–1276) or high SDI country (940.1 YLLs per 100,000; 95% UI: 815.2–1010.1) estimates. While the global and high SDI country YLLs rates for falls remained relatively stable from 1990 to 2019, Australia experienced an increase. In 1990, the global (1081 YLLs per 100,000; 95% UI: 971.3–1162.2) and high SDI countries (922 YLLs per 100,000; 95% UI: 968.5) YLLs rates for falls were significantly higher than the Australian rate.

### Leading causes of YDLs among those aged 70 and older

**Fig. 2:**
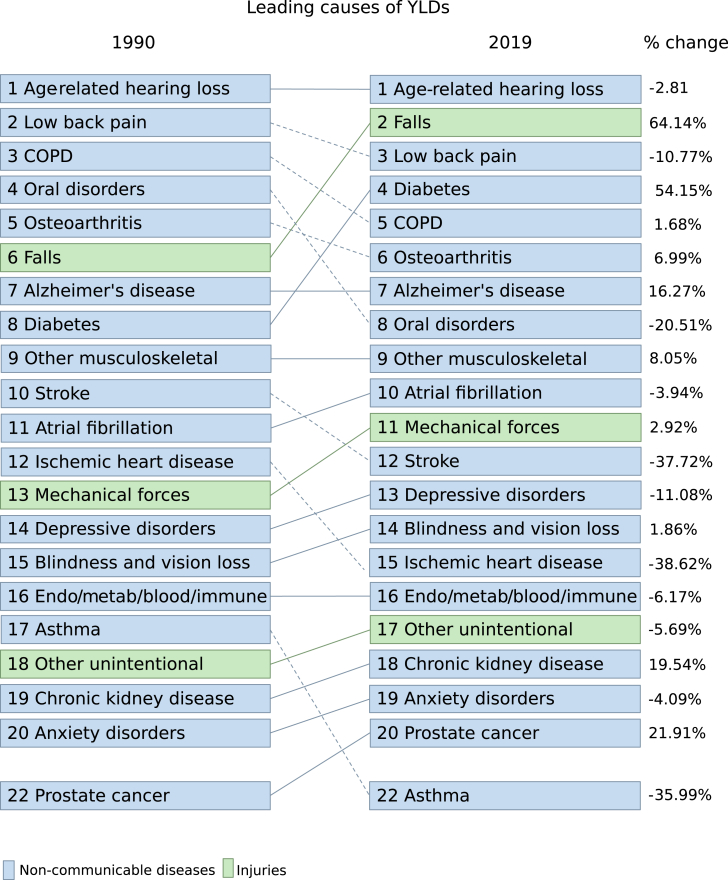
The top causes (Level 3) and % change of YLDs for ages 70 and above, both sexes combined in Australia 1990–2019. Dotted lines show a decrease in rank and solid lines show an increase in rank.

The total non-fatal burden among older adults aged 70 and above in Australia remained stable over three decades, standing at 27,913 (95% UI: 21,067–35,438) YLDs per 100,000 in 1990 and 28,596 (95% UI: 21,754–36,033) YLDs per 100,000 in 2019. Musculoskeletal disorders maintained their status as the leading group of causes for disability burden in 2019 (19% of total YLDs), followed by unintentional injuries (13.6% of total YLDs), sense organ diseases (11.7% of total YLDs), cardiovascular diseases (8.9% of total YLDs), and diabetes and kidney disease (8.8% of total YLDs), collectively contributing to 62% of total non-fatal burden (Level 2). The top 20 causes of YLDs at Level 3 for 1990 and 2019 are detailed in Fig. 2.

In 2019, falls emerged as the primary cause of YLDs among females, with 2731 YLDs per 100,000 (95% UI: 1947–3725), representing 9.4% of total female YLDs. However, there was no significant increase in the rate of YLDs attributed to falls for both males and females over the past 30 years. Age-related hearing loss, the leading cause of YLDs in males in 2019, accounted for 10.5% of total male YLDs and exhibited no significant difference from 1990 (1990: 3207 YLDs per 100,000, 95% UI: 2264–4413; 2019: 2931 YLDs per 100,000, 95% UI: 2018–4055) (Fig. 2).

Mental disorders' YLD rates among adults aged 70 and above showed no significant change over the analysis period (1990: 1,617, 95% UI: 1193–2084 YLDs per 100,000; 2019: 1,518, 95% UI: 1123–1953 YLDs per 100,000), with similar trends observed for both males and females.

### Total health loss burden for those 70 and older in Australia

In line with global trends, Australia witnessed a 25.6% reduction in the overall health burden among individuals aged 70 and older, measured in DALYs. This decline occurred over the past three decades, with figures dropping from 106,463 DALYs per 100,000 (95% UI: 99,705–11,388) in 1990 to 79,181 DALYs per 100,000 (95% UI: 72,239–86,535) in 2019. This trend was consistent for both sexes (Table 2). Notably, while there was a decrease in the health burden attributed to cardiovascular diseases (56.1%), neoplasms (13.6%), and chronic respiratory diseases (21.5%) between 1990 and 2019, these conditions remained the primary causes of disease burden for Australians aged 70 and above in 2019, collectively representing 53% of total DALYs.

**Table 2:** The 20 top sex-specific leading causes of DALYs (Level 3) in Australia from 1990 to 2019.

DALYs per 100,000										
Causes of DALYs	Females					Males				
(ranked by both sexes 2019)	1990	2000	2010	2019	% change (1990–2019)	1990	2000	2010	2019	% change (1990–2019)
Ischemic heart disease	21,694.02 (19,666–22,835)	14,284.03 (12,477–15,336)	8924.61 (7346–9829)	7099.28 (5824–7914)	−67.3∗	33,104.76	21,209.41	12,759.62	10,350.02	−68.7∗
						(31,538–34,288)	(19,773–22,170)	(11,661–13,519)	(9300–11,075)	
Alzheimer's disease and other dementias	5019.99 (2323–11,057)	5570.98 (2593–11,759)	6309.78 (2964–13,116)	6024.64 (2820–12,421)	20	2997.71 (1288–7068)	3203.51 (1398–7185)	3721.94 (1626–8308)	3853.55 (1665–8607)	28.50
Chronic obstructive pulmonary disease	4067.13 (3591–4558)	4572.50 (3932–5132)	4573.38 (3790–5255)	4436.05 (3536–5119)	9.1	10,157.97 (9352–10,870)	7861.15 (7140–8515)	6089.09 (5302–6806)	5549.91 (4740–6417)	−45.4∗
Stroke	10,168.02 (9091–10,844)	7684.56 (6715–8324)	5853.35 (4952–6437)	4927.27 (4076–5525)	−51.5∗	10,653.67 (9948–11,280)	7824.46 (7246–8298)	5247.59 (4740–5646)	4463.32 (3945–4948)	−58.1∗
Falls	2324.67 (1830–2975)	2644.44 (2093–3348)	3611.66 (2857–4545)	3795.81 (3004–4780)	63.3∗	1806.47 (1466–2257)	2194.92 (1762–2740)	3057.02 (2492–3791)	3239.78 (2624–4012)	79.3∗
Tracheal, bronchus, and lung cancer	1849.41 (1701–1967)	2188.52 (1974–2339)	2371.13 (2055–2568)	2310.88 (1979–2603)	25∗	7238.60 (6874–7564)	6122.79 (5781–6392)	5069.24 (4691–5328)	4355.00 (3951–4751)	−39.8∗
Diabetes mellitus	2581.10 (2197–3028)	3146.01 (2535–3841)	3323.87 (2673–4121)	2779.41 (2193–3478)	7.7	3255.68 (2778–3818)	3927.80 (3330–4667)	4076.50 (3372–4966)	3730.78 (2998–4662)	14.60
Age-related and other hearing loss	2287.38 (1603–3181)	2206.44 (1554–3022)	2437.65 (1713–3386)	2290.24 (1557–3205)	0.1	3207.48 (2264–4413)	2783.64 (1953–3809)	2885.52 (2049–3943)	2930.85 (2018–4055)	−8.60
Colon and rectum cancer	2567.75 (2348–2730)	2335.47 (2073–2512)	2001.25 (1707–2188)	1934.85 (1631–2184)	−24.6∗	3771.96 (3549–3988)	3429.56 (3203–3634)	2865.63 (2638–3055)	2655.40 (2317–3024)	−29.6∗
Chronic kidney disease	1618.31 (1442–1807)	1625.03 (1418–1803)	1911.42 (1612–2147)	1911.82 (1583–2199)	18.1	1848.68 (1682–2030)	1999.64 (1820–2188)	2236.18 (2004–2469)	2277.56 (2026–2546)	23.20
Atrial fibrillation and flutter	2125.00 (1703–2635)	2201.31 (1768–2700)	2242.38 (1782–2775)	2138.39 (1692–2655)	0.6	1851.61 (1398–2412)	1847.74 (1404–2376)	1917.30 (1435–2451)	1899.66 (1410–2434)	2.60
Low back pain	2276.71 (1567–3042)	2203.15 (1525–2985)	2180.60 (1529–2961)	2082.40 (1408–2877)	−8.5	2058.78 (1423–2819)	1955.02 (1360–2688)	1948.04 (1363–2626)	1799.62 (1200–2463)	−12.60
Prostate cancer	–	–	–	–	–	5480.09 (4080–6616)	5185.00 (4077–6502)	4735.38 (3889–6440)	4270.36 (3507–6119)	−22.10
Osteoarthritis	1829.06 (936–3628)	1934.88 (993–3899)	1906.98 (976–3822)	1996.84 (1031–3996)	9.2	1091.13 (555–2198)	1153.43 (588–2332)	1203.65 (615–2454)	1214.05 (621–2443)	11.30
Other musculoskeletal disorders	1489.01 (966–2130)	1671.82 (1110–2371)	1576.48 (1058–2172)	1628.02 (1054–2374)	9.3	966.24 (580–1494)	1127.08 (698–1638)	1071.97 (656–1549)	1155.96 (666–1744)	19.60
Oral disorders	1780.20 (1206–2435)	1507.06 (971–2174)	1351.56 (919–1875)	1442.19 (935–2098)	−19	1359.58 (916–1887)	1133.72 (730–1673)	1080.05 (724–1519)	1088.36 (688–1626)	−19.90
Lower respiratory infections	1142.65 (990–1245)	1373.87 (1171–1505)	1107.79 (893–1231)	1223.53 (956–1408)	7.1	1577.09 (1438–1703)	1716.73 (1557–1848)	1222.15 (1088–1321)	1265.78 (1087–1426)	−19.7∗
Parkinson's disease	701.99 (627–771)	758.99 (637–838)	789.58 (768–874)	728.68 (621–815)	3.8	1482.79 (1377–1594)	1684.44 (1538–1827)	1774.34 (1618–1926)	1651.41 (1469–1820)	11.40
Pancreatic cancer	893.85 (816–954)	944.48 (849–1008)	989.58 (847–1078)	1020.11 (856–1174)	14.1	1170.26 (1095–1240)	1117.46 (1043–1187)	1210.54 (1113–1291)	1223.90 (1072–1384)	4.60
Breast cancer	2264.64 (2065–2433)	2038.93 (1831–2203)	1939.79 (1673–2121)	1900.28 (1602–2149)	−16.1	–	–	–	–	–

Percent change (% change) of statistical significance is bolded and marked by an asterisk (∗).

Over the past three decades, while certain causes decreased their contribution to DALYs, there was a 19.7% increase in DALYs attributed to Alzheimer's disease (AD). Figures rose from 4206 DALYs per 100,000 (95% UI: 1913–9449) in 1990 to 5033 DALYs per 100,000 (95% UI: 2290–10,655) in 2019, although with wide overlapping uncertainty intervals, warranting cautious interpretation. A similar trend was observed in global and high SDI country estimates.

The most substantial increase (67.4%) in total burden over the last three decades was associated with falls, surging from 2117 DALYs per 100,000 in 1990 (95% UI: 1690–2682) to 3542. DALYs per 100,000 in 2019 (95% UI: 2838–4419), consistent for both males and females. This rise primarily stemmed from increased YLLs due to falls.

DALYs attributed to mental disorders (Level 2 cause) among Australians aged 70 and above remained stable over time, with rates of 1617 DALYs per 100,000 (95% UI: 1193–2084) in 1990 and 1518 DALYs per 100,000 (95% UI: 1123–1953) in 2019.

For a comparison of the top 20 sex specific DALYs between global estimates, Australia, composite scores of the ‘High SDI’ group of 38 countries, and selected 14 individual countries, please refer to Supplementary Figures S3 and S4.

## Discussion

The Global Burden of Diseases, Injuries, and Risk Factors Study (GBD) is the most comprehensive scientific effort to quantify fatal and non-fatal disease burden worldwide. Our analysis of Australian data shows that the health of the 70 and older age group has steadily improved over the past 30 years, as indicated by decrease in both YLL and DALY rates. Overall life expectancy is increasing, largely due to improved longevity in males and specifically driven by a reduction of YLLs attributable to cardiovascular (60%) and chronic respiratory (30.2%) diseases, and a substantial reduction in transport injuries (56.9%).

Despite an overall reduction in DALY rates, the absolute number of DALYs for this group is rapidly growing (72.4% increase from 1990 to 2019) with the increasing older population. As a word of caution for policy makers we urge to remain aware that the absolute number of DALYs, and not the DALY rates, represents the total burden of disease that the Australia's health system must manage and prepare for it to continue to increase. While there have been substantial improvements in DALYs for cardiovascular diseases, neoplasms, and chronic respiratory diseases, the burden of illness remains high. Policy targeting risk reduction, screening and evidence-based intervention is needed to optimise healthy aging and minimise personal, social and economic burden.^23^ While some environmental and lifestyle risks are on the whole decreasing, for instance smoking, others like poor air quality are worsening^24^ and require urgent coordinated intervention. Particularly, addressing inequity in access to health care and healthy lifestyles for minority groups and rural populations is critical^25^^,^^26^ to target excess modifiable burden. Taking a life course approach and designing interventions to modify risk from middle age may provide the best outcomes beyond 70 years of age.^27^ Innovation in digital monitoring, telemedicine and in home support services may contribute to future gains by enabling healthy independent living.^28^

Falls in older people are one of a major public concern due to the associated morbidity and the cost to health services. We found a large increase in DALY rates attributed to falls across 1990 to 2019 in Australia, now caught up with many other high SDI countries (see Supplementary Figures S3 and S4 for details) to match the global average. These high GBD estimates of 2% in males and 1.7% in females annual increase of DALY rates attributed to falls over the last thirty years corresponds with a report by the Australian Institute of Health and Welfare on the trends of hospitalised fall-related injuries in older people (above 65 years) from 2006 to 2007 to 2016–2017, which showed across that 10-year period, yearly fall-rates increased by 3% for males and by 2% for females. The causes of falls are multifactorial, involving both intrinsic and extrinsic factors. In particular, fall injury risk has been associated with female gender and number of physical and lifestyle-related factors, such as the presence of chronic health conditions, like stroke, having mobility problems, polypharmacy, alcohol consumption, vision loss^29^ and a low body mass index.^30^ Furthermore, it's worth noting that Musculoskeletal Disorders (MSD), which continue to be the primary cause of disability burden (accounting for 19% of total YLDs), are well-known contributors to falls.^31^ There is also a potential link between falls and use of specific drugs that impair mobility, including antipsychotics.^32^ Potential overuse and misuse of antipsychotics in older persons including those with dementia has been a long-standing concern in Australia. Starting with the 2005 ‘Dementia Initiative’, *also known as* the ‘Dementia – A National Health Priority Initiative’ (Department of Health, 2005), the Australian government has implemented a number of strategies and policies to address this issue.^33^ These interventions have produced only modest results, and a recent audit suggests that compliance with the Australian Government subsidised Pharmaceutical Benefits Scheme (PBS) guidelines, limiting antipsychotic use to last resort and for 12 weeks only, is limited.^34^ Although there was an overall increase in falls between 1990 and 2019, we do observe the YLLs and YLDs rates for falls beginning to slowly decrease around 2015, suggesting either that the impact of falls and management of complications is improving.

In examining the trends of chronic kidney disease (CKD) from 1990 to 2019 among individuals aged 70 and over, our data reveals a 44.9% increase in deaths attributed to CKD, with notable gender-specific variations. Despite this significant mortality surge, the measured burden using metrics such as DALYs, YLDs, and YYLs did not exhibit a statistically significant increase during the same period. This observation prompts a deeper exploration into potential contributing factors. Plausible explanations may encompass advancements in medical management, fostering improved access to healthcare services, or alterations in CKD diagnostic criteria.^35^ The absence of a proportional increase in burden metrics challenges our understanding of the overall impact of CKD on this demographic. In the pursuit of a comprehensive interpretation, further investigation into the complex interplay of these factors is warranted to refine our understanding and inform targeted interventions for this vulnerable population.

### Limitations

First, this study is based on GBD 2019 data and methodology, and therefore shares the limitations of the overall study, such as the major limitation of the GBD analysis of the burden of diseases and injuries being the availability of primary data. Despite having comprehensive vital registration data, Australia faces limitations in obtaining information on morbidity and health outcomes related to behavioural and metabolic risk factors. Additionally, data pertaining to inpatient and outpatient hospital admissions, health system access, and health financing were not accessible for the GBD 2019 study. Although the list of 364 causes of burden in GBD 2019 is comprehensive, some specific age-related conditions might be missing and, multimorbidity is not taken into account. While GBD provides national level estimates that are comparable with worldwide estimates, it would be highly beneficial to consider including the sub-national estimates, as well as the estimates by the socio-economic and demographic groups, such as excess mortality and decreased life expectancy of Indigenous Australians.^36^

### Conclusions

Despite population aging, the health of Australians aged 70 and above has improved over 30 years. However, their impact on healthcare, though, is growing. In 2019, the leading causes of DALYs were ischemic heart disease, Alzheimer's, COPD, stroke, and falls, with the latter experiencing the most significant increase since 1990, emphasising the escalating burden on Australia's healthcare infrastructure. Policymakers are urged to recognise the tangible burden conveyed by the total number of DALYs.

A life course approach, promoting middle-age health and addressing social and healthcare disparities, can enhance outcomes past 70, facilitated by digital healthcare. Falls policies for this group needs multifaceted strategies considering, comorbidity and medication misuse, especially antipsychotics. The non-fatal disease burden, including mental disorders, remained static from 1990 to 2019 in Australians over 70, underscoring the need for further promotion of healthy aging and reducing personal, societal, and economic impacts. Despite limitations, this study provides a pre-COVID baseline for understanding mortality and disease burden among Australians aged 70 and above, emphasising the ongoing need to promote healthy aging and address emerging healthcare challenges.

## GBD 2019 Australia Adults Over 70 Collaborators

Liliana G Ciobanu, Nadezhda V Baryshnikova, Magdalene Catharine Jawahar, Catherine G Toben, Elysia Sokolenko, Victoria Kiriaki Arnet, Isaac Yeboah Addo, Oyelola A Adegboye, Bright Opoku Ahinkorah, Khurshid Alam, Sheikh Mohammad Alif, Edward Kwabena Ameyaw, Deanna Anderlini, Blake Angell, Adnan Ansar, Anayochukwu Edward Anyasodor, Thomas Astell-Burt, Prince Atorkey, Beatriz Paulina Ayala Quintanilla, Getinet Ayano, Abraham Samuel Babu, Nasser Bagheri, Bernhard T Baune, Dinesh Bhandari, Sonu Bhaskar, Soufiane Boufous, Andrew M Briggs, Norma B Bulamu, Richard A Burns, Andre F Carvalho, Ester Cerin, Nicolas Cherbuin, Enayet Karim Chowdhury, Marita Cross, Diego De Leo, Tim Robert Driscoll, Mi Du, David Edvardsson, Kristina Edvardsson, Ferry Efendi, Aklilu Endalamaw, Nelsensius Klau Fauk, Joanne Flavel, Richard Charles Franklin, Tiffany K Gill, Bhawna Gupta, Vivek Kumar Gupta, Mohammad Hamiduzzaman, Graeme J Hankey, Simon I Hay, Jeffrey J Hebert, Delia Hendrie, Catherine L Hill, M Mamun Huda, Sheikh Mohammed Shariful Islam, Billingsley Kaambwa, Himal Kandel, Gizat M Kassie, Jessica A Kerr, Asaduzzaman Khan, M Nuruzzaman Khan, Vishnutheertha Kulkarni, Ratilal Lalloo, Long Khanh Dao Le, James Leigh, Gang Liu, Rashidul Alam Mahumud, Abdullah A Mamun, John J McGrath, Atte Meretoja, Ted R Miller, Philip B Mitchell, Ali H Mokdad, Lidia Morawska, Kehinde O Obamiro, Amy E Peden, Konrad Pesudovs, Azizur Rahman, Md Mijanur Rahman, Muhammad Aziz Rahman, Zubair Ahmed Ratan, Lal Rawal, Susan Fred Rumisha, Perminder S Sachdev, Abdul-Aziz Seidu, Saurab Sharma, Seyed Afshin Shorofi, Soraya Siabani, Ambrish Singh, Balbir Bagicha Singh, Helen Slater, Mark A Stokes, Narayan Subedi, Santosh Kumar Tadakamadla, Amanda G Thrift, Mai Thi Ngoc Tran, Corneel Vandelanotte, Ning Wang, Paul Ward, Mark Woodward, Xiaoyue Xu, Lalit Yadav, Sojib Bin Zaman, Jianrong Zhang, and Scott Richard Clark.

## Affiliations

Discipline of Psychiatry, Adelaide Medical School (L G Ciobanu PhD, E Sokolenko PhD, M C Jawahar PhD, C G Toben PhD, V K Arnet PhD, S R Clark PhD), School of Economics (N V Baryshnikova PhD), Adelaide Medical School (T K Gill PhD, Prof C L Hill MD, L Yadav PhD), School of Public Health (M Du MSc), University of Adelaide, Adelaide, SA, Australia; School of Pharmacy and Medical Sciences Quality Use of Medicines and Pharmacy Research Centre (G M Kassie PhD), University of South Australia, Adelaide, SA, Australia; Centre for Social Research in Health (I Y Addo PhD), Transport and Road Safety (TARS) Research Centre (S Boufous PhD), School of Psychiatry (Prof P B Mitchell MD), School of Public Health and Community Medicine (A E Peden PhD), School of Optometry and Vision Science (Prof K Pesudovs PhD), Faculty of Medicine and Health (S Sharma PhD), University of New South Wales, Sydney, NSW, Australia; Quality and Systems Performance Unit (I Y Addo PhD), Cancer Institute NSW, Sydney, NSW, Australia; Menzies School of Health Research (O A Adegboye PhD), Charles Darwin University, Darwin, NT, Australia; School of Public Health (B O Ahinkorah MPhil), School of Health (S Siabani PhD), University of Technology Sydney, Sydney, NSW, Australia; Murdoch Business School (K Alam PhD), Murdoch University, Perth, WA, Australia; School of Public Health and Preventive Medicine (S M Alif PhD), Department of Epidemiology and Preventative Medicine (E K Chowdhury PhD), Department of Medicine (Prof A G Thrift PhD), Monash University, Melbourne, VIC, Australia; School of Graduate Studies (E K Ameyaw MPhil), Lingnan University, Hong Kong, China; Centre for Sensorimotor Performance (D Anderlini MD), School of Public Health (A Endalamaw MSc), Institute for Social Science Research (M Huda MSc, A A Mamun PhD), School of Health and Rehabilitation Sciences (A Khan PhD), School of Dentistry (R Lalloo PhD), Queensland Brain Institute (Prof J J McGrath MD), The University of Queensland, Brisbane, QLD, Australia; Neurology Department (D Anderlini MD), Royal Brisbane and Women's Hospital, Brisbane, QLD, Australia; Institute of Global Health (B Angell PhD), University College London, London, UK; Health System Science Program (B Angell PhD), The George Institute for Global Health, Newtown, NSW, Australia; School of Nursing and Midwifery (A Ansar PhD, Prof D Edvardsson PhD, F Efendi PhD, M Rahman PhD), The Judith Lumley Centre (B Ayala Quintanilla PhD), La Trobe University, Melbourne, VIC, Australia; Special Interest Group International Health (A Ansar PhD), Public Health Association of Australia, Canberra, ACT, Australia; School of Dentistry and Medical Sciences (A E Anyasodor PhD), Charles Sturt University, Orange, NSW, Australia; School of Architecture, Design, and Planning (Prof T Astell-Burt PhD), Faculty of Medicine and Health (M Cross PhD), School of Public Health (Prof T R Driscoll PhD), Sydney Medical School (S Islam PhD), Save Sight Institute (H Kandel PhD), Department of Public Health (M Khan PhD), Asbestos Diseases Research Institute (J Leigh MD), NHMRC Clinical Trials Centre (R A Mahumud PhD), The Daffodil Centre (M Rahman PhD), School of Veterinary Science (B B Singh PhD), University of Sydney, Sydney, NSW, Australia; School of Medicine and Public Health (P Atorkey MPhil), University of Newcastle, Newcastle, NSW, Australia; Hunter New England Population Health, Wallsend, NSW, Australia (P Atorkey MPhil); San Martin de Porres University, Lima, Peru (B Ayala Quintanilla PhD); School of Indigenous Studies (G Ayano MSc), University of Western Australia, Perth, WA, Australia; School of Public Health (G Ayano MSc, E K Chowdhury PhD, D Hendrie PhD, T R Miller PhD), School of Physiotherapy and Exercise Science (Prof A M Briggs PhD, Prof H Slater PhD), Curtin University, Perth, WA, Australia; Department of Physiotherapy (A S Babu PhD), Manipal Academy of Higher Education, Manipal, India; Research School of Population Health (N Bagheri PhD, R A Burns PhD, Prof N Cherbuin PhD), Australian National University, Canberra, ACT, Australia; Health Research Institute (N Bagheri PhD), University of Canberra, Canberra, ACT, Australia; Department of Psychiatry (Prof B T Baune PhD), University of Münster, Münster, Germany; Department of Psychiatry (Prof B T Baune PhD), Melbourne Medical School, Melbourne, VIC, Australia; School of Nursing and Midwifery (D Bhandari PhD), Monash University, Clayton, VIC, Australia; Public Health Research Laboratory (D Bhandari PhD), Central Department of Public Health (N Subedi MPH), Tribhuvan University, Kathmandu, Nepal; Global Health Neurology Lab (S Bhaskar PhD), NSW Brain Clot Bank, Sydney, NSW, Australia; Department of Neurology and Neurophysiology (S Bhaskar PhD), South West Sydney Local Heath District and Liverpool Hospital, Sydney, NSW, Australia; Flinders Health and Medical Research Institute (N B Bulamu PhD), Health Economics Unit (B Kaambwa PhD), College of Medicine and Public Health (B Kaambwa PhD), Department of Nursing and Health Sciences (S Shorofi PhD), Flinders University, Adelaide, SA, Australia; IMPACT Strategic Research Center (the Institute for Mental and Physical Health and Clinical Translation) (A F Carvalho MD), Deakin University, Geelong, VIC, Australia; Mary MacKillop Institute for Health Research (Prof E Cerin PhD), Australian Catholic University, Melbourne, VIC, Australia; School of Public Health (Prof E Cerin PhD), University of Hong Kong, Hong Kong, China; Global Alliance for Musculoskeletal Health, Sydney, NSW, Australia (M Cross PhD); Australian Institute for Suicide Research and Prevention (Prof D De Leo DSc), Griffith University, Mount Gravatt, QLD, Australia; Department of Periodontology (M Du MSc), Shandong University, Jinan, China; Department of Nursing (Prof D Edvardsson PhD), Umeå University, Umea, Sweden; College of Science, Health and Engineering (K Edvardsson PhD), La Trobe University, Bundoora, VIC, Australia; Department of Community Health Nursing (F Efendi PhD), Universitas Airlangga (Airlangga University), Surabaya, Indonesia; Department of Pediatrics and Child Health Nursing (A Endalamaw MSc), Bahir Dar University, Bahir Dar, Ethiopia; Centre for Health Policy Research (Prof P Ward PhD), Torrens University Australia, Adelaide, SA, Australia (N K Fauk MSc); Institute of Resource Governance and Social Change, Kupang, Indonesia (N K Fauk MSc); School of Social Sciences (J Flavel PhD), Stretton Health Equity, Adelaide, SA, Australia; College of Public Health, Medical, and Veterinary Sciences (Prof R C Franklin PhD, A E Peden PhD), College of Public Health, Medical and Veterinary Sciences (A Seidu MPhil), James Cook University, Townsville, QLD, Australia (K O Obamiro PhD); Department of Public Health (B Gupta PhD), Torrens University Australia, Melbourne, VIC, Australia; Faculty of Medicine Health and Human Sciences (Prof V K Gupta PhD), Macquarie University, Sydney, NSW, Australia; Faculty of Health (M Hamiduzzaman PhD), Southern Cross University, Bilinga, QLD, Australia; Centre for Neuromuscular and Neurological Disorders (Prof G J Hankey MD), The University of Western Australia, Perth, WA, Australia; Perron Institute for Neurological and Translational Science, Perth, WA, Australia (Prof G J Hankey MD); Institute for Health Metrics and Evaluation (Prof S I Hay FMedSci, A H Mokdad PhD), Department of Health Metrics Sciences, School of Medicine (Prof S I Hay FMedSci, A H Mokdad PhD), University of Washington, Seattle, WA, USA; Faculty of Kinesiology (Prof J J Hebert PhD), University of New Brunswick, Fredericton, NB, Canada; School of Allied Health (Prof J J Hebert PhD), Murdoch University, Murdoch, WA, Australia; Rheumatology Department (Prof C L Hill MD), The Queen Elizabeth Hospital, Woodville, SA, Australia; Institute for Physical Activity and Nutrition (S Islam PhD), Department of Psychology (M A Stokes PhD), Deakin University, Burwood, VIC, Australia; Sydney Eye Hospital (H Kandel PhD), South Eastern Sydney Local Health District, Sydney, NSW, Australia; Centre for Adolescent Health (J A Kerr PhD), Murdoch Childrens Research Institute, Parkville, VIC, Australia; Department of Psychological Medicine (J A Kerr PhD), University of Otago, Christchurch, New Zealand; Population Science Department (M Khan PhD), Jatiya Kabi Kazi Nazrul Islam University, Mymensingh, Bangladesh; Department of Medicine (V Kulkarni MS), Queensland Health, Brisbane, QLD, Australia; Health Economics Division (L K D Le PhD), Monash University, Burwood, VIC, Australia; School of Life Sciences (G Liu PhD), University of Technology Sydney, Ultimo, NSW, Australia; National Centre for Register-based Research (Prof J J McGrath MD), Aarhus University, Aarhus, Denmark; Neurology Unit (A Meretoja MD), Helsinki University Hospital, Helsinki, Finland; School of Health Sciences (A Meretoja MD), Department of General Practice (J Zhang MD), University of Melbourne, Melbourne, VIC, Australia; Pacific Institute for Research & Evaluation, Calverton, MD, USA (T R Miller PhD); International Laboratory for Air Quality and Health (Prof L Morawska PhD), School of Public Health and Social Work (M T N Tran PhD, N Wang PhD), Queensland University of Technology, Brisbane, QLD, Australia; Data Mining Research Unit (DaMRA) (A Rahman PhD), Charles Sturt University, Wagga Wagga, NSW, Australia; School of Medicine and Public Health (M Rahman PhD), The University of Newcastle, Wollongong, NSW, Australia; Institute of Health and Wellbeing (M Rahman PhD), Federation University Australia, Berwick, VIC, Australia; Department of Biomedical Engineering (Z Ratan MSc), Khulna University of Engineering and Technology, Khulna, Bangladesh; School of Health and Society (Z Ratan MSc), University of Wollongong, Wollongong, NSW, Australia; School of Health, Medical and Applied Sciences (L Rawal PhD), CQ University, Sydney, NSW, Australia; The Malaria Atlas Project (S F Rumisha PhD), Telethon Kids Institute, Perth, WA, Australia; Department of Health Statistics (S F Rumisha PhD), National Institute for Medical Research, Dar es Salaam, Tanzania; School of Psychiatry (Prof P S Sachdev MD), The George Institute (Prof M Woodward PhD), School of Population Health (X Xu PhD), University of New South Wales, Kensington, NSW, Australia; Neuropsychiatric Institute (Prof P S Sachdev MD), Prince of Wales Hospital, Randwick, NSW, Australia; Department of Population and Health (A Seidu MPhil), University of Cape Coast, Cape Coast, Ghana; Department of Physiotherapy (S Sharma PhD), Kathmandu University, Dhulikhel, Nepal; Department of Medical-Surgical Nursing (S Shorofi PhD), Mazandaran University of Medical Sciences, Sari, Iran; Department of Health Education and Health Promotion (S Siabani PhD), Kermanshah University of Medical Sciences, Kermanshah, Iran; Menzies Institute for Medical Research (A Singh MTech), University of Tasmania, Hobart, TAS, Australia; School of Public Health & Zoonoses (B B Singh PhD), Guru Angad Dev Veterinary & Animal Sciences University, Ludhiana, India; School of Exercise and Nutrition Sciences (N Subedi MPH), Deakin University, Melbourne, VIC, Australia; School of Dentistry and Oral Health (S K Tadakamadla PhD), Griffith University, Gold Coast, QLD, Australia; Health Informatics Department (M T N Tran PhD), Hanoi Medical University, Ha Noi, Viet Nam; Department of Medical and Applied Sciences (Prof C Vandelanotte PhD), Central Queensland University, Rockhampton, QLD, Australia; National Center for Chronic and Noncommunicable Disease Control and Prevention (N Wang PhD), Chinese Center for Disease Control and Prevention, Beijing, China; The George Institute for Global Health (Prof M Woodward PhD), University of New South Wales, Camperdown, NSW, Australia; Cardiovascular Program (X Xu PhD), The George Institute for Global Health, Sydney, NSW, Australia; Australian Institute of Health Innovation (L Yadav PhD), Macquarie University, Macquarie Park, NSW, Australia; Department of Health Sciences (S Zaman MSc), James Madison University, Harrisonburg, VA, USA; Victorian Comprehensive Cancer Centre, Melbourne, VIC, Australia (J Zhang MD)

## Contributors

LGC, NVB, MCJ, CGT, ES, and VKA prepared the first draft. LGC, NVB, MCJ, CGT, ES, VKA, and SRC analysed the data and edited the first draft and final versions of the manuscript. LGC, NVB, and SRC finalised all drafts, and approved the final version of the manuscript. All other authors provided data, developed models, reviewed results, provided guidance on methodology, or reviewed the manuscript, and approved the final version of the manuscript. Please see appendix for more detailed information about individual author contributions to the research, divided into the following categories: providing data or critical feedback on data sources, developing methods or computational machinery; providing critical feedback on methods or results; drafting the work or revising it critically for important intellectual content; and managing the estimation or publications process.

## Data sharing statement

The data is publicly available at: https://vizhub.healthdata.org/gbd-compare/.

## Declaration of interests

SC was supported by Janssen-Cilag Australia and Lundbeck Otsuka grants paid to institution (The University of Adelaide). B Angell reports a grant from the National Health and Medical Research Council (Australia) (Investigator Grant–GNT GNT2010055), outside the submitted work. P Atorkey reports infrastructure support for the present manuscript from the Australian College of Applied Professions, Discipline of Psychological Sciences, and the School of Medicine and Public Health, University of Newcastle. S Bhaskar reports grants or contracts from Japan Society for the Promotion of Science (JSPS), Japanese Ministry of Education, Culture, Sports, Science and Technology (MEXT) for the Grant-in-Aid for Scientific Research (KAKENHI) and from the JSPS and the Australian Academy of Science for a JSPS International Fellowship; leadership or fiduciary roles in other board, society, committee or advocacy group, paid or unpaid, with Rotary District 9675 as District Chair, Diversity, Equity & Inclusion, Global Health & Migration Hub Community, Global Health Hub Germany, Berlin, Germany as Chair and Manager, PLOS One, BMC Neurology, Frontiers in Neurology, Frontiers in Stroke, Frontiers in Public Health & BMC Medical Research Methodology as Editorial Board Member, and College of Reviewers, Canadian Institutes of Health Research (CIHR), Government of Canada as Member; all outside the submitted work. A M Briggs reports research grants paid to their institution from AO Alliance, Asia Pacific League of Associations for Rheumatology, Australian Rheumatology Association, Pan American League of Associations for Rheumatology, World Federation of Chiropractic, Australian Government Department of Health Grant, National Health and Medical Research Council (Australia) Medical Research Future Fund Grant, Western Australian Government Department of Health Grant, Bone and Joint Decade Foundation (Sweden), Institute for Bone and Joint Research (Australia), Canadian Memorial Chiropractic College, Arthritis and Osteoporosis Western Australia, and Arthritis Australia; consulting frees as a senior consultant to World Health Organization for technical advice and technical products development related to ageing and musculoskeletal health, from OneSpace Health for services as a consultant physiotherapist, and from WorkSafe Victoria for work in reviewing management pathways for injured workers in the state of Victoria; honoraria for lectures, presentations, speakers bureaus, manuscript writing or educational events from the American College of Rheumatology for a presentation in 2022 and the Austrian Institute for Health Technology Assessment for independent reviews of their policy documents in 2021; support for attending meetings and/or travel from WHO for travel to work at the Headquarters in Geneva for technical work, the University of Otago to attend the New Zealand Osteoarthritis Summit in 2023 (Dunedin, New Zealand), and the World Federation of Chiropractic to attend their scientific meeting in 2023 (Gold Coast, Australia); leadership or fiduciary roles in other board, society, committee or advocacy group, unpaid, as a member of the International Coordinating Council for the Global Alliance for Musculoskeletal Health (G-MUSC); all outside the submitted work. S R Clark reports grants or contracts from Janssen Cilag Australia Investigator Initiated paid to the University of Adelaide; payment or honoraria for lectures, presentations, speakers bureaus, manuscript writing or educational events from Lundbeck Otsuka for speakers fees and manuscript writing paid to the University of Adelaide; participation on an Advisory Board with Lundbeck Otsuka paid to the University of Adelaide; all outside the submitted work. R C Franklin reports Support for attending meetings and/or travel from the Australasian College of Tropical Medicine (ACTM)—Tropical Medicine and Travel Medicine Conference 2022, 2023, and the International Society of Travel Medicine (ISTM)—Travel Medicine Conference, Basel 2023; leadership or fiduciary roles in other board, society, committee or advocacy group, paid or unpaid, with Kidsafe as President/Director, Farmsafe as Director, Auschem as Director, PHAA Injury Prevention SIG Convener, ISASH as Governance Committee member, and ACTM as Vice President; all outside the submitted work. S M S Islam reports support for the present manuscript from the National Health and Medical Research Council (Australia) through an Investigator Grant and the Heart Foundation of Australia through a Vanguard Grant. P B Mitchell reports payment or honoraria for lectures, presentations, speakers bureaus, manuscript writing or educational events from Janssen (Australia) outside the submitted work. A E Peden reports support for the present manuscript from the National Health and Medical Research Council (Australia) (Grant Number: APP2009306). P S Sachdev reports grants or contracts paid to their institution from National Health and Medical Research Council (Australia) and National Institutes of Health (USA); payment or honoraria for lectures, presentations, speakers bureaus, manuscript writing or educational events from Alkem Labs for a lecture as part of the Frontiers of Psychiatry 2023 seminar, Mumbai, India, June 2023; participation on a Data Safety Monitoring Board or Advisory Board with Biogen Australia on the Medical Advisory Committee in 2020 and 2021 and Roche Australia on the Medical Advisory Committee in 2022; leadership or fiduciary roles in other board, society, committee or advocacy group, unpaid, with VASCOG Society Executive Committee and the World Psychiatric Association Planning Committee; all outside the submitted work. S Sharma reports support for the present manuscript paid to their institution from the John J. Bonica Postdoctoral Fellowship from the International Association for the Study of Pain (2021–2023); payment outside the submitted work for an online lecture on pain to compensate for time in January 2023; support outside the submitted work from the International Association for the Study of Pain to offset travel for the World Congress on Pain in Toronto in 2022. H Slater reports grants or contracts paid to their institution from Australian Government, Department of Health Grant, Medical Research Future Fund Grant, Western Australian Government Department of Health Grant, Bone and Joint Decade Foundation (Sweden) Grant, Curtin University (Australia), Institute for Bone and Joint Research (Australia), Canadian Memorial Chiropractic College (Canada): Grants; support for attending meetings and/or travel from the Australian Pain Society; all outside the submitted work. A G Thrift reports grants for research projects paid to their institution from the National Health & Medical Research Council (Australia), Heart Foundation (Australia), and Stroke Foundation (Australia); a leadership or fiduciary role in other board, society, committee or advocacy group, unpaid, with the Stroke Foundation Board (Australia); all outside the submitted work.

## Appendix A Supplementary data

Supplementary Figures

GBD 2019 data sources

Supplementary Table S3

Authors & Affiliations_AUS70.docx
