# Supplementary material for: Pre-COVID life expectancy, mortality, and burden of diseases for adults 70 years and older in Australia: a systematic analysis for the Global Burden of Disease 2019 Study
Source: Lancet Reg Health West Pac. 2024 Jun 5;47:101092. doi: 10.1016/j.lanwpc.2024.101092 (PMC11190477; doi:10.1016/j.lanwpc.2024.101092)
Supplement: Supplementary Figures [file mmc1.docx]

**Supplementary Figure 1.** Top 20 level 3 causes of deaths (per 100,000, females, 70 and above years) for Australia and 14 selected high SDI countries in 2019.


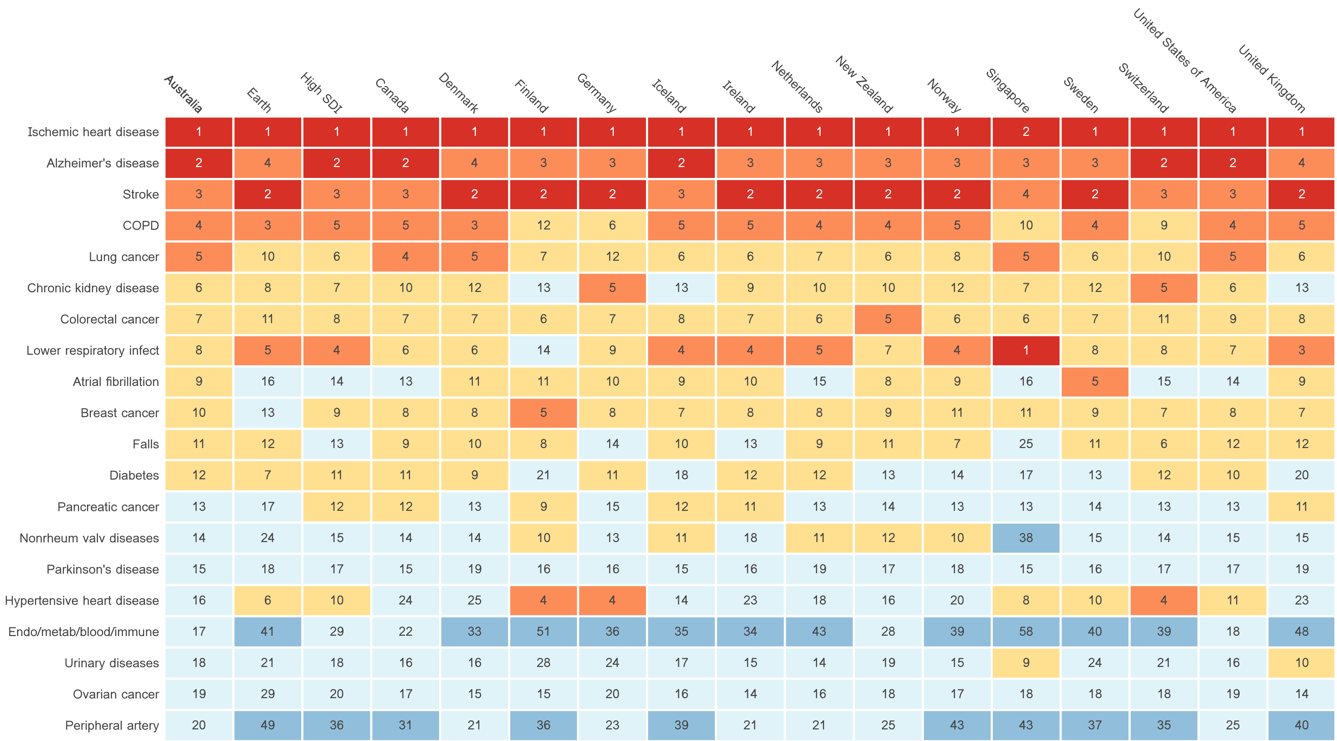


Note: The 'High SDI' average was calculated based on selected countries by applying the SDI quintile cutoff values of 0.805129 and 1 in 2019 (the quintiles determined by GBD). Therefore, the high SDI group includes the following 38 countries: Andorra, Australia, Austria, Belgium, Canada, Czech Republic, Cyprus, England, Estonia, Finland, France, Germany, Iceland, Ireland, Japan, Latvia, Lithuania, Luxembourg, Monaco, Netherlands, New Zealand, Norway, Puerto Rico, Qatar, Russia, San Marino, Saudi Arabia, Singapore, Slovakia, Slovenia, South Korea, Sweden, Switzerland, Taiwan (province of China), UAE, UK, and USA. The selection of 14 individual countries presented here for comparison follows [1].

**Supplementary Figure 2.** Top 20 level 3 causes of deaths (per 100,000, males, 70 and above years) for Australia and 14 selected high SDI countries in 2019.


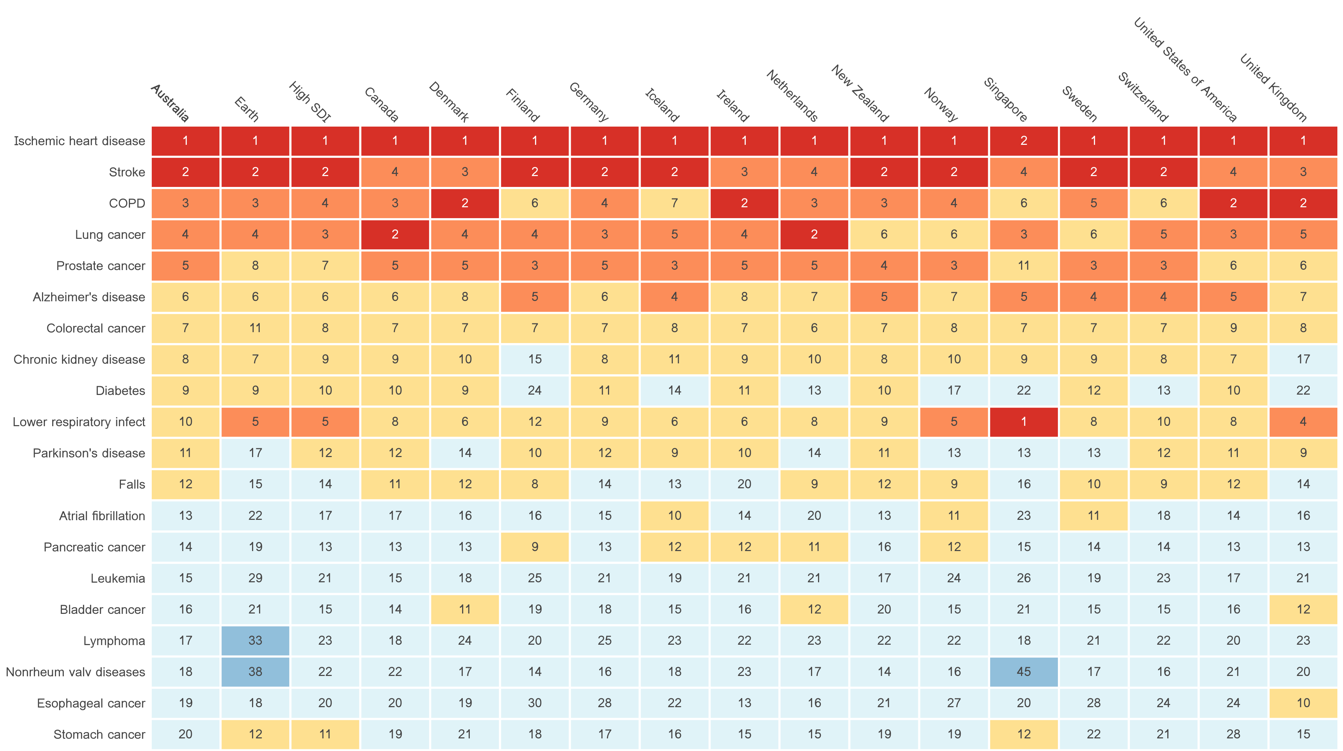


Note: The 'High SDI' average was calculated based on selected countries by applying the SDI quintile cutoff values of 0.805129 and 1 in 2019 (the quintiles determined by GBD). Therefore, the high SDI group includes the following 38 countries: Andorra, Australia, Austria, Belgium, Canada, Czech Republic, Cyprus, England, Estonia, Finland, France, Germany, Iceland, Ireland, Japan, Latvia, Lithuania, Luxembourg, Monaco, Netherlands, New Zealand, Norway, Puerto Rico, Qatar, Russia, San Marino, Saudi Arabia, Singapore, Slovakia, Slovenia, South Korea, Sweden, Switzerland, Taiwan (province of China), UAE, UK, and USA. The selection of 14 individual countries presented here for comparison follows [1].

**Supplementary Figure 3.** Top 20 level 3 causes of DALYs (per 100,000, females, 70 and above years) for Australia and 14 selected high SDI countries in 2019.**
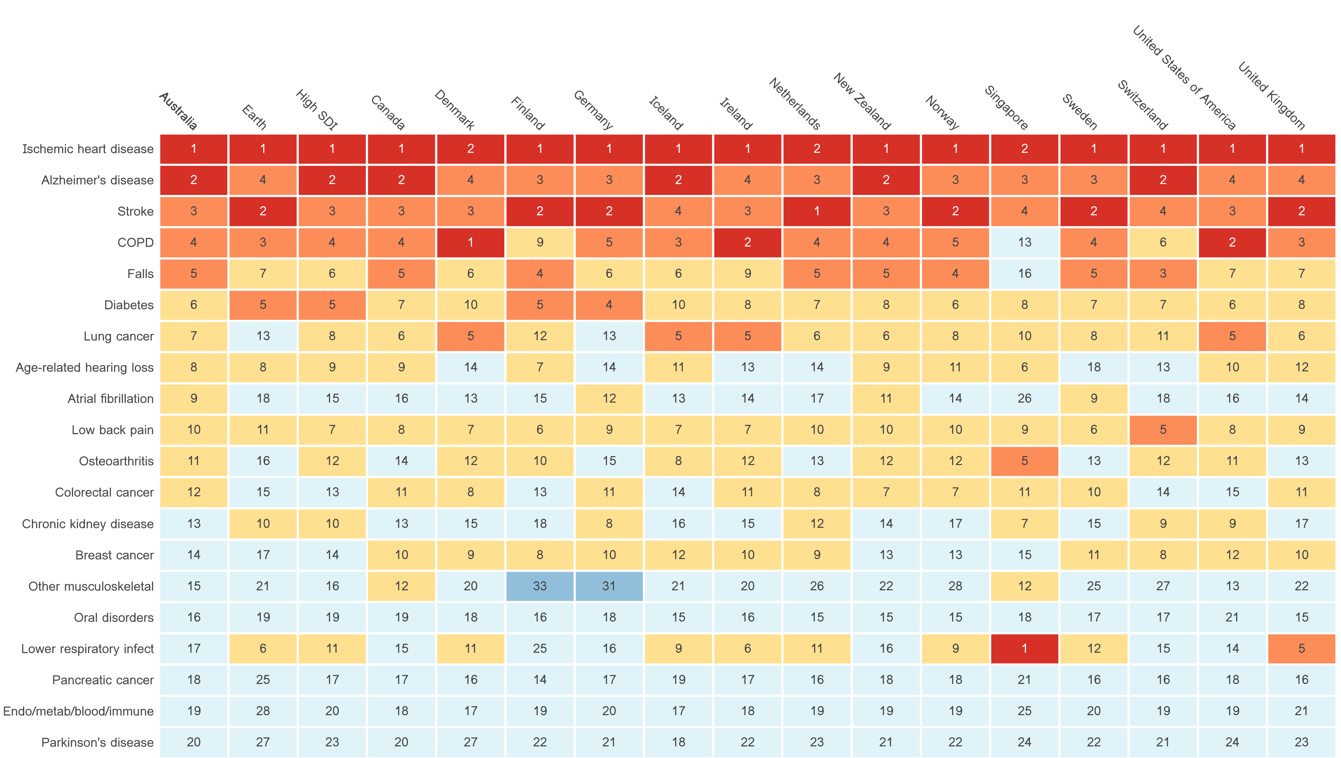
**

Note: The 'High SDI' average was calculated based on selected countries by applying the SDI quintile cutoff values of 0.805129 and 1 in 2019 (the quintiles determined by GBD). Therefore, the high SDI group includes the following 38 countries: Andorra, Australia, Austria, Belgium, Canada, Czech Republic, Cyprus, England, Estonia, Finland, France, Germany, Iceland, Ireland, Japan, Latvia, Lithuania, Luxembourg, Monaco, Netherlands, New Zealand, Norway, Puerto Rico, Qatar, Russia, San Marino, Saudi Arabia, Singapore, Slovakia, Slovenia, South Korea, Sweden, Switzerland, Taiwan (province of China), UAE, UK, and USA. The selection of 14 individual countries presented here for comparison follows [1].

**Supplementary Figure 4.** Top 20 level 3 causes of DALYs (per 100,000, males, 70 and above years) for Australia and 14 selected high SDI countries in 2019.


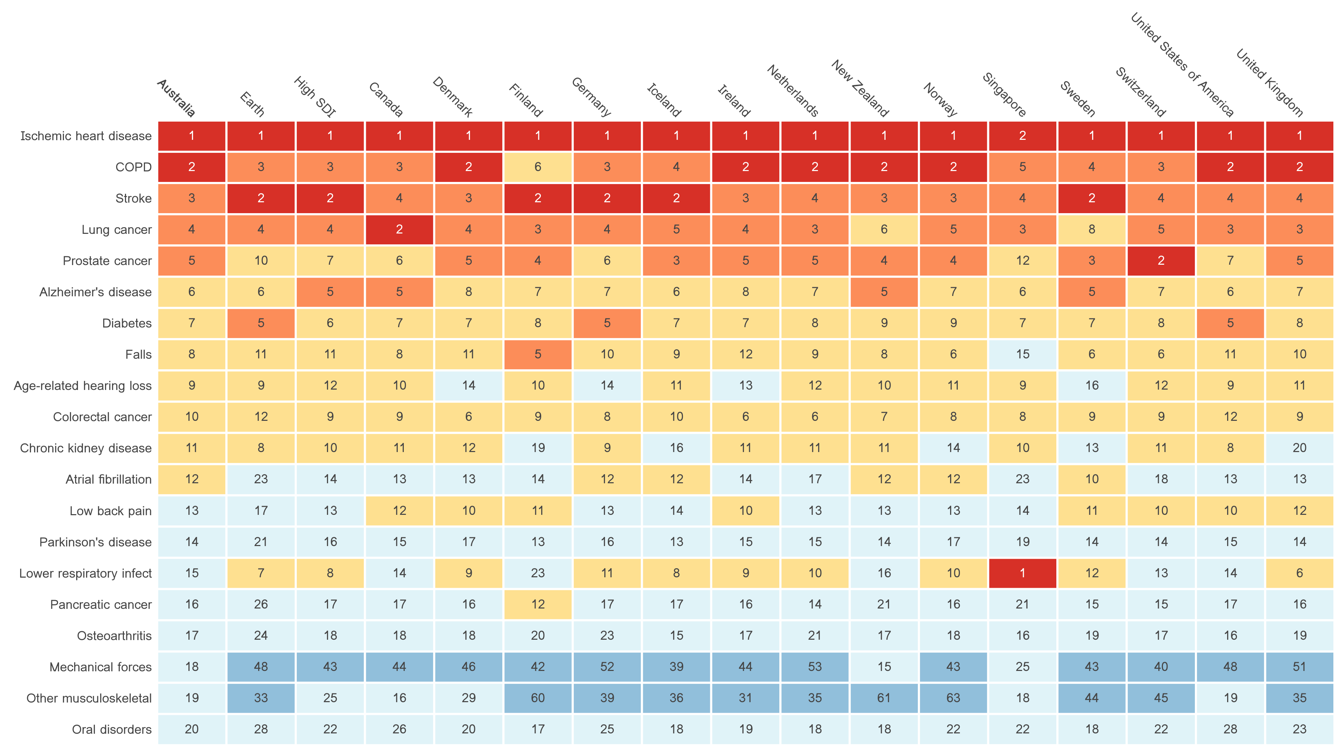


Note: The 'High SDI' average was calculated based on selected countries by applying the SDI quintile cutoff values of 0.805129 and 1 in 2019 (the quintiles determined by GBD). Therefore, the high SDI group includes the following 38 countries: Andorra, Australia, Austria, Belgium, Canada, Czech Republic, Cyprus, England, Estonia, Finland, France, Germany, Iceland, Ireland, Japan, Latvia, Lithuania, Luxembourg, Monaco, Netherlands, New Zealand, Norway, Puerto Rico, Qatar, Russia, San Marino, Saudi Arabia, Singapore, Slovakia, Slovenia, South Korea, Sweden, Switzerland, Taiwan (province of China), UAE, UK, and USA. The selection of 14 individual countries presented here for comparison follows [1].

**References:**

1. Islam, S.M.S., et al., *The burden and trend of diseases and their risk factors in Australia, 1990&#x2013;2019: a systematic analysis for the Global Burden of Disease Study 2019.* The Lancet Public Health, 2023. **8**(8): p. e585-e599.
