# Supplementary material for: Pre-COVID life expectancy, mortality, and burden of diseases for adults 70 years and older in Australia: a systematic analysis for the Global Burden of Disease 2019 Study
Source: Lancet Reg Health West Pac. 2024 Jun 5;47:101092. doi: 10.1016/j.lanwpc.2024.101092 (PMC11190477; doi:10.1016/j.lanwpc.2024.101092)
Supplement: Supplementary Table S3 [file mmc4.docx]

**Supplementary Table 3.** How causes contributed to change in life expectancy (LE) in Australia (for both sexes)

LE in 1990: 77 years

LE in 2019: 82.9 years

1990-2019 change +5.9

| **Decreased LE** | **Years** | **Increased LE** | **Years** |
| --- | --- | --- | --- |
| Substance use | -0.1 | Cardiovascular diseases | +3.5 |
| Omitted values greater than -0.5 | -0 | Neoplasms | +0.9 |
| **Total years lost** | -0.1 | Transport injuries | +0.4 |
|  |  | Chronic respiratory | +0.3 |
|  |  | Other non-communicable | +0.2 |
|  |  | Maternal & neonatal | +0.1 |
|  |  | Digestive diseases | +0.1 |
|  |  | Self-harm and violence | +0.1 |
|  |  | Unintentional injuries | +0.1 |
|  |  | Respiratory infections & TB | +0.1 |
|  |  | HIV/AIDS & STIs | +0.1 |
|  |  | Omitted values greater than -0.5 | +0.1 |
|  |  | **Total years gained** | +6 |
