# Supplementary material for: Pre-COVID life expectancy, mortality, and burden of diseases for adults 70 years and older in Australia: a systematic analysis for the Global Burden of Disease 2019 Study
Source: Lancet Reg Health West Pac. 2024 Jun 5;47:101092. doi: 10.1016/j.lanwpc.2024.101092 (PMC11190477; doi:10.1016/j.lanwpc.2024.101092)
Supplement: Authors & Affiliations_AUS70.docx [file mmc5.docx]

# GBD 2019 Australia Adults Over 70 Collaborators

Liliana G Ciobanu, Nadezhda V Baryshnikova, Magdalene Catharine Jawahar, Catherine G Toben, Elysia Sokolenko, Victoria Kiriaki Arnet, Isaac Yeboah Addo, Oyelola A Adegboye, Bright Opoku Ahinkorah, Khurshid Alam, Sheikh Mohammad Alif, Edward Kwabena Ameyaw, Deanna Anderlini, Blake Angell, Adnan Ansar, Anayochukwu Edward Anyasodor, Thomas Astell-Burt, Prince Atorkey, Beatriz Paulina Ayala Quintanilla, Getinet Ayano, Abraham Samuel Babu, Nasser Bagheri, Bernhard T Baune, Dinesh Bhandari, Sonu Bhaskar, Soufiane Boufous, Andrew M Briggs, Norma B Bulamu, Richard A Burns, Andre F Carvalho, Ester Cerin, Nicolas Cherbuin, Enayet Karim Chowdhury, Marita Cross, Diego De Leo, Tim Robert Driscoll, Mi Du, David Edvardsson, Kristina Edvardsson, Ferry Efendi, Aklilu Endalamaw, Nelsensius Klau Fauk, Joanne Flavel, Richard Charles Franklin, Tiffany K Gill, Bhawna Gupta, Vivek Kumar Gupta, Mohammad Hamiduzzaman, Graeme J Hankey, Simon I Hay, Jeffrey J Hebert, Delia Hendrie, Catherine L Hill, M Mamun Huda, Sheikh Mohammed Shariful Islam, Billingsley Kaambwa, Himal Kandel, Gizat M Kassie, Jessica A Kerr, Asaduzzaman Khan, M Nuruzzaman Khan, Vishnutheertha Kulkarni, Ratilal Lalloo, Long Khanh Dao Le, James Leigh, Gang Liu, Rashidul Alam Mahumud, Abdullah A Mamun, John J McGrath, Atte Meretoja, Ted R Miller, Philip B Mitchell, Ali H Mokdad, Lidia Morawska, Kehinde O Obamiro, Amy E Peden, Konrad Pesudovs, Azizur Rahman, Md Mijanur Rahman, Muhammad Aziz Rahman, Zubair Ahmed Ratan, Lal Rawal, Susan Fred Rumisha, Perminder S Sachdev, Abdul-Aziz Seidu, Saurab Sharma, Seyed Afshin Shorofi, Soraya Siabani, Ambrish Singh, Balbir Bagicha Singh, Helen Slater, Mark A Stokes, Narayan Subedi, Santosh Kumar Tadakamadla, Amanda G Thrift, Mai Thi Ngoc Tran, Corneel Vandelanotte, Ning Wang, Paul Ward, Mark Woodward, Xiaoyue Xu, Lalit Yadav, Sojib Bin Zaman, Jianrong Zhang, and Scott Richard Clark.

# Affiliations

Discipline of Psychiatry, Adelaide Medical School (L G Ciobanu PhD, E Sokolenko PhD, M C Jawahar PhD, C G Toben PhD, V K Arnet PhD, S R Clark PhD), School of Economics (N V Baryshnikova PhD), Adelaide Medical School (T K Gill PhD, Prof C L Hill MD, L Yadav PhD), School of Public Health (M Du MSc), University of Adelaide, Adelaide, SA, Australia; School of Pharmacy and Medical Sciences, Quality Use of Medicines and Pharmacy Research Centre (G M Kassie PhD), University of South Australia, Adelaide, SA, Australia; Centre for Social Research in Health (I Y Addo PhD), Transport and Road Safety (TARS) Research Centre (S Boufous PhD), School of Psychiatry (Prof P B Mitchell MD), School of Public Health and Community Medicine (A E Peden PhD), School of Optometry and Vision Science (Prof K Pesudovs PhD), Faculty of Medicine and Health (S Sharma PhD), University of New South Wales, Sydney, NSW, Australia; Quality and Systems Performance Unit (I Y Addo PhD), Cancer Institute NSW, Sydney, NSW, Australia; Menzies School of Health Research (O A Adegboye PhD), Charles Darwin University, Darwin, NT, Australia; School of Public Health (B O Ahinkorah MPhil), School of Health (S Siabani PhD), University of Technology Sydney, Sydney, NSW, Australia; Murdoch Business School (K Alam PhD), Murdoch University, Perth, WA, Australia; School of Public Health and Preventive Medicine (S M Alif PhD), Department of Epidemiology and Preventative Medicine (E K Chowdhury PhD), Department of Medicine (Prof A G Thrift PhD), Monash University, Melbourne, VIC, Australia; School of Graduate Studies (E K Ameyaw MPhil), Lingnan University, Hong Kong, China; Centre for Sensorimotor Performance (D Anderlini MD), School of Public Health (A Endalamaw MSc), Institute for Social Science Research (M Huda MSc, A A Mamun PhD), School of Health and Rehabilitation Sciences (A Khan PhD), School of Dentistry (R Lalloo PhD), Queensland Brain Institute (Prof J J McGrath MD), The University of Queensland, Brisbane, QLD, Australia; Neurology Department (D Anderlini MD), Royal Brisbane and Women's Hospital, Brisbane, QLD, Australia; Institute of Global Health (B Angell PhD), University College London, London, UK; Health System Science Program (B Angell PhD), The George Institute for Global Health, Newtown, NSW, Australia; School of Nursing and Midwifery (A Ansar PhD, Prof D Edvardsson PhD, F Efendi PhD, M Rahman PhD), The Judith Lumley Centre (B Ayala Quintanilla PhD), La Trobe University, Melbourne, VIC, Australia; Special Interest Group International Health (A Ansar PhD), Public Health Association of Australia, Canberra, ACT, Australia; School of Dentistry and Medical Sciences (A E Anyasodor PhD), Charles Sturt University, Orange, NSW, Australia; School of Architecture, Design, and Planning (Prof T Astell-Burt PhD), Faculty of Medicine and Health (M Cross PhD), School of Public Health (Prof T R Driscoll PhD), Sydney Medical School (S Islam PhD), Save Sight Institute (H Kandel PhD), Department of Public Health (M Khan PhD), Asbestos Diseases Research Institute (J Leigh MD), NHMRC Clinical Trials Centre (R A Mahumud PhD), The Daffodil Centre (M Rahman PhD), School of Veterinary Science (B B Singh PhD), University of Sydney, Sydney, NSW, Australia; School of Medicine and Public Health (P Atorkey MPhil), University of Newcastle, Newcastle, NSW, Australia; Hunter New England Population Health, Wallsend, NSW, Australia (P Atorkey MPhil); San Martin de Porres University, Lima, Peru (B Ayala Quintanilla PhD); School of Indigenous Studies (G Ayano MSc), University of Western Australia, Perth, WA, Australia; School of Public Health (G Ayano MSc, E K Chowdhury PhD, D Hendrie PhD, T R Miller PhD), School of Physiotherapy and Exercise Science (Prof A M Briggs PhD, Prof H Slater PhD), Curtin University, Perth, WA, Australia; Department of Physiotherapy (A S Babu PhD), Manipal Academy of Higher Education, Manipal, India; Research School of Population Health (N Bagheri PhD, R A Burns PhD, Prof N Cherbuin PhD), Australian National University, Canberra, ACT, Australia; Health Research Institute (N Bagheri PhD), University of Canberra, Canberra, ACT, Australia; Department of Psychiatry (Prof B T Baune PhD), University of Münster, Münster, Germany; Department of Psychiatry (Prof B T Baune PhD), Melbourne Medical School, Melbourne, VIC, Australia; School of Nursing and Midwifery (D Bhandari PhD), Monash University, Clayton, VIC, Australia; Public Health Research Laboratory (D Bhandari PhD), Central Department of Public Health (N Subedi MPH), Tribhuvan University, Kathmandu, Nepal; Global Health Neurology Lab (S Bhaskar PhD), NSW Brain Clot Bank, Sydney, NSW, Australia; Department of Neurology and Neurophysiology (S Bhaskar PhD), South West Sydney Local Heath District and Liverpool Hospital, Sydney, NSW, Australia; Flinders Health and Medical Research Institute (N B Bulamu PhD), Health Economics Unit (B Kaambwa PhD), College of Medicine and Public Health (B Kaambwa PhD), Department of Nursing and Health Sciences (S Shorofi PhD), Flinders University, Adelaide, SA, Australia; IMPACT Strategic Research Center (the Institute for Mental and Physical Health and Clinical Translation) (A F Carvalho MD), Deakin University, Geelong, VIC, Australia; Mary MacKillop Institute for Health Research (Prof E Cerin PhD), Australian Catholic University, Melbourne, VIC, Australia; School of Public Health (Prof E Cerin PhD), University of Hong Kong, Hong Kong, China; Global Alliance for Musculoskeletal Health, Sydney, NSW, Australia (M Cross PhD); Australian Institute for Suicide Research and Prevention (Prof D De Leo DSc), Griffith University, Mount Gravatt, QLD, Australia; Department of Periodontology (M Du MSc), Shandong University, Jinan, China; Department of Nursing (Prof D Edvardsson PhD), Umeå University, Umea, Sweden; College of Science, Health and Engineering (K Edvardsson PhD), La Trobe University, Bundoora, VIC, Australia; Department of Community Health Nursing (F Efendi PhD), Universitas Airlangga (Airlangga University), Surabaya, Indonesia; Department of Pediatrics and Child Health Nursing (A Endalamaw MSc), Bahir Dar University, Bahir Dar, Ethiopia; Centre for Health Policy Research (Prof P Ward PhD), Torrens University Australia, Adelaide, SA, Australia (N K Fauk MSc); Institute of Resource Governance and Social Change, Kupang, Indonesia (N K Fauk MSc); School of Social Sciences (J Flavel PhD), Stretton Health Equity, Adelaide, SA, Australia; College of Public Health, Medical, and Veterinary Sciences (Prof R C Franklin PhD, A E Peden PhD), College of Public Health, Medical and Veterinary Sciences (A Seidu MPhil), James Cook University, Townsville, QLD, Australia (K O Obamiro PhD); Department of Public Health (B Gupta PhD), Torrens University Australia, Melbourne, VIC, Australia; Faculty of Medicine Health and Human Sciences (Prof V K Gupta PhD), Macquarie University, Sydney, NSW, Australia; Faculty of Health (M Hamiduzzaman PhD), Southern Cross University, Bilinga, QLD, Australia; Centre for Neuromuscular and Neurological Disorders (Prof G J Hankey MD), The University of Western Australia, Perth, WA, Australia; Perron Institute for Neurological and Translational Science, Perth, WA, Australia (Prof G J Hankey MD); Institute for Health Metrics and Evaluation (Prof S I Hay FMedSci, A H Mokdad PhD), Department of Health Metrics Sciences, School of Medicine (Prof S I Hay FMedSci, A H Mokdad PhD), University of Washington, Seattle, WA, USA; Faculty of Kinesiology (Prof J J Hebert PhD), University of New Brunswick, Fredericton, NB, Canada; School of Allied Health (Prof J J Hebert PhD), Murdoch University, Murdoch, WA, Australia; Rheumatology Department (Prof C L Hill MD), The Queen Elizabeth Hospital, Woodville, SA, Australia; Institute for Physical Activity and Nutrition (S Islam PhD), Department of Psychology (M A Stokes PhD), Deakin University, Burwood, VIC, Australia; Sydney Eye Hospital (H Kandel PhD), South Eastern Sydney Local Health District, Sydney, NSW, Australia; Centre for Adolescent Health (J A Kerr PhD), Murdoch Childrens Research Institute, Parkville, VIC, Australia; Department of Psychological Medicine (J A Kerr PhD), University of Otago, Christchurch, New Zealand; Population Science Department (M Khan PhD), Jatiya Kabi Kazi Nazrul Islam University, Mymensingh, Bangladesh; Department of Medicine (V Kulkarni MS), Queensland Health, Brisbane, QLD, Australia; Health Economics Division (L K D Le PhD), Monash University, Burwood, VIC, Australia; School of Life Sciences (G Liu PhD), University of Technology Sydney, Ultimo, NSW, Australia; National Centre for Register-based Research (Prof J J McGrath MD), Aarhus University, Aarhus, Denmark; Neurology Unit (A Meretoja MD), Helsinki University Hospital, Helsinki, Finland; School of Health Sciences (A Meretoja MD), Department of General Practice (J Zhang MD), University of Melbourne, Melbourne, VIC, Australia; Pacific Institute for Research & Evaluation, Calverton, MD, USA (T R Miller PhD); International Laboratory for Air Quality and Health (Prof L Morawska PhD), School of Public Health and Social Work (M T N Tran PhD, N Wang PhD), Queensland University of Technology, Brisbane, QLD, Australia; Data Mining Research Unit (DaMRA) (A Rahman PhD), Charles Sturt University, Wagga Wagga, NSW, Australia; School of Medicine and Public Health (M Rahman PhD), The University of Newcastle, Wollongong, NSW, Australia; Institute of Health and Wellbeing (M Rahman PhD), Federation University Australia, Berwick, VIC, Australia; Department of Biomedical Engineering (Z Ratan MSc), Khulna University of Engineering and Technology, Khulna, Bangladesh; School of Health and Society (Z Ratan MSc), University of Wollongong, Wollongong, NSW, Australia; School of Health, Medical and Applied Sciences (L Rawal PhD), CQ University, Sydney, NSW, Australia; The Malaria Atlas Project (S F Rumisha PhD), Telethon Kids Institute, Perth, WA, Australia; Department of Health Statistics (S F Rumisha PhD), National Institute for Medical Research, Dar es Salaam, Tanzania; School of Psychiatry (Prof P S Sachdev MD), The George Institute (Prof M Woodward PhD), School of Population Health (X Xu PhD), University of New South Wales, Kensington, NSW, Australia; Neuropsychiatric Institute (Prof P S Sachdev MD), Prince of Wales Hospital, Randwick, NSW, Australia; Department of Population and Health (A Seidu MPhil), University of Cape Coast, Cape Coast, Ghana; Department of Physiotherapy (S Sharma PhD), Kathmandu University, Dhulikhel, Nepal; Department of Medical-Surgical Nursing (S Shorofi PhD), Mazandaran University of Medical Sciences, Sari, Iran; Department of Health Education and Health Promotion (S Siabani PhD), Kermanshah University of Medical Sciences, Kermanshah, Iran; Menzies Institute for Medical Research (A Singh MTech), University of Tasmania, Hobart, TAS, Australia; School of Public Health & Zoonoses (B B Singh PhD), Guru Angad Dev Veterinary & Animal Sciences University, Ludhiana, India; School of Exercise and Nutrition Sciences (N Subedi MPH), Deakin University, Melbourne, VIC, Australia; School of Dentistry and Oral Health (S K Tadakamadla PhD), Griffith University, Gold Coast, QLD, Australia; Health Informatics Department (M T N Tran PhD), Hanoi Medical University, Ha Noi, Viet Nam; Department of Medical and Applied Sciences (Prof C Vandelanotte PhD), Central Queensland University, Rockhampton, QLD, Australia; National Center for Chronic and Noncommunicable Disease Control and Prevention (N Wang PhD), Chinese Center for Disease Control and Prevention, Beijing, China; The George Institute for Global Health (Prof M Woodward PhD), University of New South Wales, Camperdown, NSW, Australia; Cardiovascular Program (X Xu PhD), The George Institute for Global Health, Sydney, NSW, Australia; Australian Institute of Health Innovation (L Yadav PhD), Macquarie University, Macquarie Park, NSW, Australia; Department of Health Sciences (S Zaman MSc), James Madison University, Harrisonburg, VA, USA; Victorian Comprehensive Cancer Centre, Melbourne, VIC, Australia (J Zhang MD)

Declarations

B Angell reports a grant from the National Health and Medical Research Council (Australia) (Investigator Grant - GNT GNT2010055), outside the submitted work. P Atorkey reports infrastructure support for the present manuscript from the Australian College of Applied Professions, Discipline of Psychological Sciences, and the School of Medicine and Public Health, University of Newcastle. S Bhaskar reports grants or contracts from Japan Society for the Promotion of Science (JSPS), Japanese Ministry of Education, Culture, Sports, Science and Technology (MEXT) for the Grant-in-Aid for Scientific Research (KAKENHI) and from the JSPS and the Australian Academy of Science for a JSPS International Fellowship; leadership or fiduciary roles in other board, society, committee or advocacy group, paid or unpaid, with Rotary District 9675 as District Chair, Diversity, Equity & Inclusion, Global Health & Migration Hub Community, Global Health Hub Germany, Berlin, Germany as Chair and Manager, PLOS One, BMC Neurology, Frontiers in Neurology, Frontiers in Stroke, Frontiers in Public Health & BMC Medical Research Methodology as Editorial Board Member, and College of Reviewers, Canadian Institutes of Health Research (CIHR), Government of Canada as Member; all outside the submitted work. A M Briggs reports research grants paid to their institution from AO Alliance, Asia Pacific League of Associations for Rheumatology, Australian Rheumatology Association, Pan American League of Associations for Rheumatology, World Federation of Chiropractic, Australian Government Department of Health Grant, National Health and Medical Research Council (Australia) Medical Research Future Fund Grant, Western Australian Government Department of Health Grant, Bone and Joint Decade Foundation (Sweden), Institute for Bone and Joint Research (Australia), Canadian Memorial Chiropractic College, Arthritis and Osteoporosis Western Australia, and Arthritis Australia; consulting frees as a senior consultant to World Health Organization for technical advice and technical products development related to ageing and musculoskeletal health, from OneSpace Health for services as a consultant physiotherapist, and from WorkSafe Victoria for work in reviewing management pathways for injured workers in the state of Victoria; honoraria for lectures, presentations, speakers bureaus, manuscript writing or educational events from the American College of Rheumatology for a presentation in 2022 and the Austrian Institute for Health Technology Assessment for independent reviews of their policy documents in 2021; support for attending meetings and/or travel from WHO for travel to work at the Headquarters in Geneva for technical work, the University of Otago to attend the New Zealand Osteoarthritis Summit in 2023 (Dunedin, New Zealand), and the World Federation of Chiropractic to attend their scientific meeting in 2023 (Gold Coast, Australia); leadership or fiduciary roles in other board, society, committee or advocacy group, unpaid, as a member of the International Coordinating Council for the Global Alliance for Musculoskeletal Health (G-MUSC); all outside the submitted work. S R Clark reports grants or contracts from Janssen Cilag Australia Investigator Initiated paid to the University of Adelaide; payment or honoraria for lectures, presentations, speakers bureaus, manuscript writing or educational events from Lundbeck Otsuka for speakers fees and manuscript writing paid to the University of Adelaide; participation on an Advisory Board with Lundbeck Otsuka paid to the University of Adelaide; all outside the submitted work. R C Franklin reports Support for attending meetings and/or travel from the Australasian College of Tropical Medicine (ACTM) – Tropical Medicine and Travel Medicine Conference 2022, 2023, and the International Society of Travel Medicine (ISTM) – Travel Medicine Conference, Basel 2023; leadership or fiduciary roles in other board, society, committee or advocacy group, paid or unpaid, with Kidsafe as President/Director, Farmsafe as Director, Auschem as Director, PHAA Injury Prevention SIG Convener, ISASH as Governance Committee member, and ACTM as Vice President; all outside the submitted work. S M S Islam reports support for the present manuscript from the National Health and Medical Research Council (Australia) through an Investigator Grant and the Heart Foundation of Australia through a Vanguard Grant. P B Mitchell reports payment or honoraria for lectures, presentations, speakers bureaus, manuscript writing or educational events from Janssen (Australia) outside the submitted work. A E Peden reports support for the present manuscript from the National Health and Medical Research Council (Australia) (Grant Number: APP2009306). P S Sachdev reports grants or contracts paid to their institution from National Health and Medical Research Council (Australia) and National Institutes of Health (USA); payment or honoraria for lectures, presentations, speakers bureaus, manuscript writing or educational events from Alkem Labs for a lecture as part of the Frontiers of Psychiatry 2023 seminar, Mumbai, India, June 2023; participation on a Data Safety Monitoring Board or Advisory Board with Biogen Australia on the Medical Advisory Committee in 2020 and 2021 and Roche Australia on the Medical Advisory Committee in 2022; leadership or fiduciary roles in other board, society, committee or advocacy group, unpaid, with VASCOG Society Executive Committee and the World Psychiatric Association Planning Committee; all outside the submitted work. S Sharma reports support for the present manuscript paid to their institution from the John J. Bonica Postdoctoral Fellowship from the International Association for the Study of Pain (2021-2023); payment outside the submitted work for an online lecture on pain to compensate for time in January 2023; support outside the submitted work from the International Association for the Study of Pain to offset travel for the World Congress on Pain in Toronto in 2022. H Slater reports grants or contracts paid to their institution from Australian Government, Department of Health Grant, Medical Research Future Fund Grant, Western Australian Government Department of Health Grant, Bone and Joint Decade Foundation (Sweden) Grant, Curtin University (Australia), Institute for Bone and Joint Research (Australia), Canadian Memorial Chiropractic College (Canada): Grants; support for attending meetings and/or travel from the Australian Pain Society; all outside the submitted work. A G Thrift reports grants for research projects paid to their institution from the National Health & Medical Research Council (Australia), Heart Foundation (Australia), and Stroke Foundation (Australia); a leadership or fiduciary role in other board, society, committee or advocacy group, unpaid, with the Stroke Foundation Board (Australia); all outside the submitted work.

Authors’ Contributions

Providing data or critical feedback on data sources

Oyelola A Adegboye, Bright Opoku Ahinkorah, Sheikh Mohammad Alif, Edward Kwabena Ameyaw, Deanna Anderlini, Prince Atorkey, Beatriz Paulina Ayala Quintanilla, Getinet Ayano, Dinesh Bhandari, Sonu Bhaskar, Soufiane Boufous, Scott Richard Clark, Tim Robert Driscoll, Mohammad Hamiduzzaman, Simon I Hay, Jeffrey J Hebert, Sheikh Mohammed Shariful Islam, Billingsley Kaambwa, Himal Kandel, M Nuruzzaman Khan, James Leigh, Gang Liu, Rashidul Alam Mahumud, Abdullah A Mamun, Atte Meretoja, Ted R Miller, Ali H Mokdad, Kehinde O Obamiro, Azizur Rahman, Zubair Ahmed Ratan, Lal Rawal, Susan Fred Rumisha, Soraya Siabani, Ambrish Singh, Mark A Stokes, Mai Thi Ngoc Tran, and Mark Woodward.

### Developing methods or computational machinery

Tim Robert Driscoll, Simon I Hay, James Leigh, Rashidul Alam Mahumud, Ali H Mokdad, and Susan Fred Rumisha.

### Providing critical feedback on methods or results

Isaac Yeboah Addo, Oyelola A Adegboye, Bright Opoku Ahinkorah, Khurshid Alam, Sheikh Mohammad Alif, Edward Kwabena Ameyaw, Deanna Anderlini, Blake Angell, Anayochukwu Edward Anyasodor, Thomas Astell-Burt, Prince Atorkey, Beatriz Paulina Ayala Quintanilla, Getinet Ayano, Abraham Samuel Babu, Nasser Bagheri, Bernhard T Baune, Dinesh Bhandari, Sonu Bhaskar, Andrew M Briggs, Norma B Bulamu, Richard A Burns, Andre F Carvalho, Ester Cerin, Nicolas Cherbuin, Enayet Karim Chowdhury, Liliana G Ciobanu, Scott Richard Clark, Marita Cross, Mi Du, David Edvardsson, Kristina Edvardsson, Ferry Efendi, Aklilu Endalamaw, Joanne Flavel, Richard Charles Franklin, Tiffany K Gill, Bhawna Gupta, Vivek Kumar Gupta, Mohammad Hamiduzzaman, Simon I Hay, Delia Hendrie, Catherine L Hill, M Mamun Huda, Sheikh Mohammed Shariful Islam, Billingsley Kaambwa, Himal Kandel, Gizat M Kassie, Jessica A Kerr, Asaduzzaman Khan, M Nuruzzaman Khan, Vishnutheertha Kulkarni, Long Khanh Dao Le, Gang Liu, Rashidul Alam Mahumud, Abdullah A Mamun, Atte Meretoja, Ted R Miller, Philip B Mitchell, Ali H Mokdad, Lidia Morawska, Kehinde O Obamiro, Amy E Peden, Konrad Pesudovs, Azizur Rahman, Md Mijanur Rahman, Muhammad Aziz Rahman, Zubair Ahmed Ratan, Lal Rawal, Abdul-Aziz Seidu, Saurab Sharma, Seyed Afshin Shorofi, Soraya Siabani, Ambrish Singh, Balbir Bagicha Singh, Mark A Stokes, Santosh Kumar Tadakamadla, Amanda G Thrift, Mai Thi Ngoc Tran, Corneel Vandelanotte, Ning Wang, Paul Ward, Mark Woodward, Xiaoyue Xu, Sojib Bin Zaman, and Jianrong Zhang.

### Drafting the work or revising it critically for important intellectual content

Isaac Yeboah Addo, Oyelola A Adegboye, Bright Opoku Ahinkorah, Khurshid Alam, Deanna Anderlini, Blake Angell, Adnan Ansar, Anayochukwu Edward Anyasodor, Victoria Kiriaki Arnet, Prince Atorkey, Beatriz Paulina Ayala Quintanilla, Abraham Samuel Babu, Nadezhda V Baryshnikova, Bernhard T Baune, Dinesh Bhandari, Sonu Bhaskar, Soufiane Boufous, Norma B Bulamu, Richard A Burns, Andre F Carvalho, Ester Cerin, Nicolas Cherbuin, Liliana G Ciobanu, Scott Richard Clark, Marita Cross, Diego De Leo, Mi Du, David Edvardsson, Nelsensius Klau Fauk, Joanne Flavel, Tiffany K Gill, Bhawna Gupta, Mohammad Hamiduzzaman, Graeme J Hankey, Jeffrey J Hebert, M Mamun Huda, Sheikh Mohammed Shariful Islam, Magdalene Catharine Jawahar, Himal Kandel, Gizat M Kassie, Jessica A Kerr, M Nuruzzaman Khan, Vishnutheertha Kulkarni, Ratilal Lalloo, James Leigh, Rashidul Alam Mahumud, Abdullah A Mamun, John J McGrath, Atte Meretoja, Ted R Miller, Philip B Mitchell, Ali H Mokdad, Kehinde O Obamiro, Amy E Peden, Konrad Pesudovs, Azizur Rahman, Lal Rawal, Susan Fred Rumisha, Perminder S Sachdev, Saurab Sharma, Seyed Afshin Shorofi, Balbir Bagicha Singh, Helen Slater, Elysia Sokolenko, Mark A Stokes, Narayan Subedi, Santosh Kumar Tadakamadla, Amanda G Thrift, Catherine G Toben, Mai Thi Ngoc Tran, Corneel Vandelanotte, Paul Ward, Mark Woodward, Lalit Yadav, Sojib Bin Zaman, and Jianrong Zhang.

### Managing the estimation or publications process

Simon I Hay, Rashidul Alam Mahumud, and Ali H Mokdad.
